# The Impact of Retirement on Cardiovascular Disease and Its Risk Factors: A Systematic Review of Longitudinal Studies

**DOI:** 10.1093/geront/gnz062

**Published:** 2019-05-15

**Authors:** Baowen Xue, Jenny Head, Anne McMunn

**Affiliations:** Department of Epidemiology and Public Health, University College London, UK

**Keywords:** Cardiovascular disease, Retired, Metabolic risk factors, Smoking, Drinking

## Abstract

**Background and Objectives:**

People are now spending longer in retirement than ever before and retirement has been found to influence health. This study systematically reviewed the impact of retirement on cardiovascular disease (CVD) and its risk factors (metabolic risk factors, blood biomarkers, physical activity, smoking, drinking, and diet).

**Research Design and Methods:**

Longitudinal studies published in Medline, Embase, Social Science Citation Index, PsycINFO, and Social Policy and Practice were searched. No language restrictions were applied if there was an English abstract. Eighty-two longitudinal studies were included after critical appraisals.

**Results:**

Studies in the United States often found no significant effect of retirement on CVD, while studies in European countries, except France, showed a detrimental effect of retirement on CVD. Results from the United States and several European countries consistently show that retirement increase adiposity measures among those retired from physically demanding jobs. For diabetes and hypertension, five out of nine studies suggest no effect of retirement. Retirement has been repeatedly linked to increasing leisure-time physical activity but may reduce work- and transport-related physical activity in turn. Most studies showed that retirement either decreased smoking or had no effect on smoking. The evidence did not show a clear conclusion on drinking. Only a few studies have assessed the impact on diet and blood biomarkers.

**Discussion and Implications:**

Effect of retirement varies according to the health outcomes studied and country of the study population. Policy concerning extending the retirement age needs to focus on ensuring they are suited to the individual.

Global population aging has led to shrinking ratios of workers to pensioners and people are now spending longer in retirement than ever before. Government policy in many industrialized countries is to raise state pension ages (SPA). The potential health effects of retirement are largely neglected in the political debate. In contrast, there is a growing academic literature on the health effects of retirement.

Although empirical studies have demonstrated the effect of retirement on health outcomes, evidence on the impact of retirement on cardiovascular disease (CVD), however, is ambiguous and has not yet been systematically summarized. For instance, Behncke (2012) found that retirees had a higher risk of being diagnosed with CVD than working people. However, Westerlund and colleagues (2010) showed that retirement had no effect on CVD while Moon, Glymour, Subramanian, Avendaño, and Kawachi (2012) suggested that CVD incidence increased in the first year after retirement, but this effect was weaker from the second-year postretirement. CVD is the greatest cause of death globally and has imposed a large burden in many countries (WHO, 2017) and the development of CVD might be complicated. Retirement may influence the development of CVD through behavioral pathways, such as changes in smoking, alcohol consumption, physical activity, and diet. These health behaviors themselves are risk factors for CVD (Mendis, Puska, & Norrving, 2011), and can affect other CVD metabolic risk factors, such as diabetes and hypertension (Roerecke et al., 2017; Schwingshackl, Missbach, König, & Hoffmann, 2015). Furthermore, retirees’ CVD risk may also be influenced by retirement-related changes in mental and physical health (Elderon & Whooley, 2013; Williams et al., 2012).

The aim of this study is to provide a systematic literature review, which summarizes the available evidence on the effects of retirement on CVD and its risk factors among the older population. We did not limit the age range of the study population due to different retirement ages among different countries. This review contributes to literature by, (a) filling an important gap in the literature by systematically summarizing the currently ambiguous research on the effect of retirement on CVD and (b) providing policymakers insights into the possible impact of retirement on CVD that may aggravate or alleviate pressures on the healthcare system.

## Methods

### Search Strategy

Published articles were identified by searching Medline, Embase, Social Science Citation Index, PsycINFO, and Social Policy and Practice. All databases were searched from their inception to January 2019. No language restrictions were applied if there was an English abstract. Indexing terms in combination with text words were used in searching (see Supplementary Table S1). The reference list of the eligible papers was hand searched.

### Inclusion and Exclusion Criteria

Included studies had to meet all the following criteria: (a) types of participants are the general population, irrespective of gender, race, or ethnicity; (b) exposure of interest is retirement, regardless of whether it was normal, early, old age retirement; (c) outcomes of interest included CVD and CVD risk factors; and (d) study design is longitudinal. In this review, the operational definition of retirement was not restricted, which could be self-reports of economic activities as retired, being eligible for a public pension, or stopping work at the SPA. Definitions of CVD could be any diseases of the heart and circulation except for congenital heart disease. High blood pressure, high cholesterol, diabetes, and being overweight or obesity/central obesity were all considered as metabolic risk factors of CVD. Physical inactivity, smoking, alcohol drinking and poor diet were considered as behavioral risk factors of CVD. High-density lipoprotein (sometimes called “good cholesterol” and can lower the risk of CVD), low-density lipoprotein (sometimes called “bad cholesterol” and can raise the risk of CVD), total cholesterol, glycated hemoglobin (reflects time-averaged blood glucose during the previous 8–12 weeks, and it is often used as a diagnostic test for diabetes), C-reactive protein (a sign of inflammation and potential risk factor for CVD), and any other inflammatory biomarkers were considered as blood biomarkers.

Studies were excluded if they met one or more of the following criteria: (a) does not evaluate the relationship between retirement and CVD or CVD risk factors; (b) CVD or CVD risk factors are the determinants of retirement, rather than the effects of retirement; (c) exposure is disability retirement; (d) studies among patient populations (e.g., survivors of stroke) or people in special occupations (e.g., athletes); (v) retirement is an effect modifier rather than an exposure; (vi) study on congenital heart disease or nonmodifiable CVD risk factors, such as family history or genes; (vii) non-English abstract; and (viii) small sample size (less than 50).

The first author initially screened the titles, keywords, and abstracts of selected articles and excluded those that were out of scope. Then, the first author and last author independently appraised the identified papers to determine their suitability. Critical appraisal skills program (CASP) appraisal checklists were applied. Disagreements were resolved in discussion with the second author.

### Collation, Summarizing, and Synthesis

The first author extracted data on the study population. The information extracted for each study included study design, sample size, outcome type, analytic method, and findings of the included studies (Supplementary Table S2).

## Results

**Figure 1.**
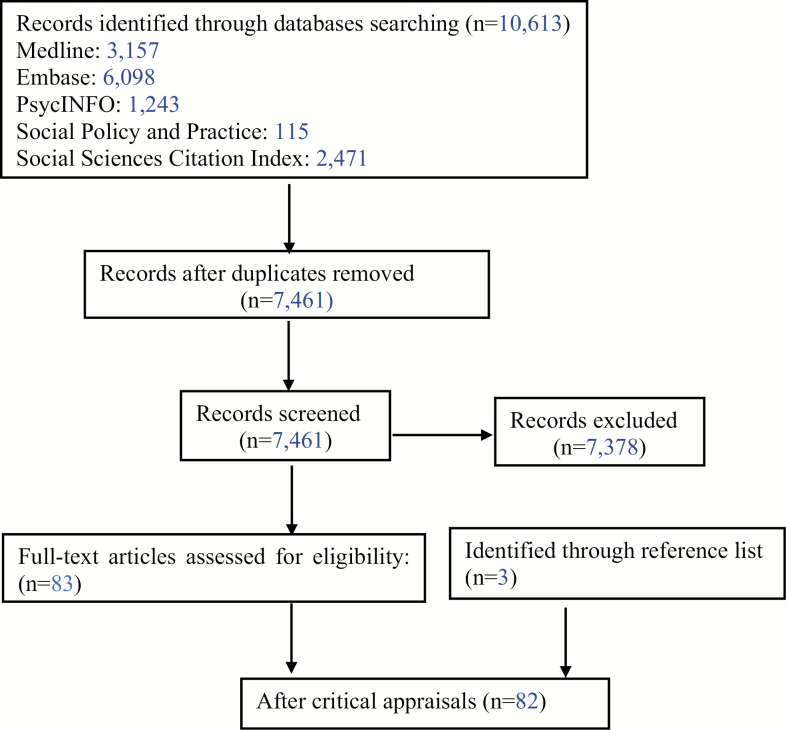
Study flow diagram.

Figure 1 shows the study flow diagram according to PRISMA guidelines (Moher, Liberati, Tetzlaff, & Altman, 2009) and a PRISMA checklist is included in Supplementary Material. After removing duplicates, database searches identified a total of 7,461 titles. After critical appraisals, a total of 82 longitudinal studies were included in this review. Most studies were excluded because they used retirement surveys (e.g., Health and Retirement Study [HRS]) but did not talk about a relevant topic.

### Retirement and CVD

Fourteen longitudinal studies assessed the association between retirement and CVD (Supplementary Table S2a). Most of the included studies were conducted in the United States and European countries. Longitudinal studies in the United States showed no significant effect of retirement on CVD incidence, at least in the long term. There were four studies from the United States, which were all based on the HRS, a representative longitudinal survey of Americans over the age of 50, although different analytic methods were applied. To elaborate, Dupre, George, Liu, and Peterson (2012) used survival analyses and they did not find any significant differences between retired and working people regarding myocardial infarction incidence. Dave, Rashad, and Spasojevic (2008) applied fixed effects models to take account of unobserved individual characteristics and reported that retirement was not associated with the incidence of heart disease and stroke once samples were restricted to those consistently insured in all waves. Coe and Lindeboom (2008) used an early retirement window as the instrumental variables (IV) and again did not find any significant effects of early retirement on myocardial infarction either in the short run (within 2 years) or long run (within 4 years). Moon et al. (2012) echoed the ideas of short- and long-term effects of retirement and, using survival analyses, suggested that the risk of CVD occurring within the first year of retirement was modestly increased, but this effect was weaker from the second-year postretirement.

Unlike the studies in the United States, most European longitudinal studies reported detrimental effects of retirement on CVD. Morris, Cook, and Shaper (1994) stated that men who retired earlier than 60 years old for reasons other than illness were more likely to die from circulatory disease during 5.5 years’ follow-up in the British Regional Heart Study. Increased fatal and nonfatal CVD incidence was also found to be associated with retirement in Denmark (Olesen, Rugulies, Rod, & Bonde, 2014), Greece (Bamia, Trichopoulou, & Trichopoulos, 2008), Italy (Petrelli, Gnavi, Marinacci, & Costa, 2006), and the Netherlands (Méjean et al., 2013). Considering CVD may be a determinant of exit from work, it is possible that the above associations between retirement and CVD have been overestimated due to the healthy worker effect. However, Behncke (2012) used the SPA as an IV for retirement in the English Longitudinal Study of Ageing (ELSA) and found that retirees had a higher risk of being diagnosed with CVD than working people. One exception is France, as none of the French studies reported significant associations between retirement and CVD. Westerlund and colleagues (2009) used the French GAZEL study to calculate the cumulative prevalence of self-reported CVD from 7 years before to 7 years after retirement. This piecewise method showed that the CVD prevalence increased with age and without a break in the trend around or after retirement, indicating no effect of retirement. Another longitudinal French study conducted in the early 1980s did not show any significant association between retirement and CVD either (Vallery-Masson et al., 1981)

In summary, studies in the United States often found no significant effect of retirement on CVD, while research in European countries (except for France) showed consistently increased CVD events among retirees. No clear pattern emerged in other regions due to the small number of relevant studies (*n* = 2).

### Retirement and CVD Metabolic Risk Factors

Fourteen studies tested the effect of retirement on BMI (Supplementary Table S2b) but conflicting results were found. One study showed that weight gain for British adults who entered retirement was equivalent to those who remained employed, suggesting no effect of retirement (Monsivais, Martin, & Suhrcke, 2015). The other British longitudinal study showed that noncontinuously employed men were more likely to either gain or lose weight, whereas weight was stable among the continuously employed men (Morris, Cook, & Shaper, 1992). This study compared a broad category of noncontinuously employed rather than retirement, which may lead to bias in the results. Syse, Veenstra, Furunes, Mykletun, and Solem (2017) found that retirees in Norway were more likely to report an increase in weight loss than employed people. Using data from several countries, Nishimura, Oikawa, and Motegi (2018) found that BMI increased after retirement in the United States and Japan, but did not change in England, Demark, France, Germany, Switzerland, and Korea. No association between retirement and BMI was found among participants of the China Health and Nutrition Survey (Xue, Head, & McMunn, 2017).

Several studies suggested that preretirement occupation modifies the effect of retirement on BMI. Four longitudinal studies based on the HRS study consistently showed that retirement was associated with higher adiposity measures among those retired from physically demanding jobs but not in other jobs (Chung, Domino, & Stearns, 2009; Forman-Hoffman et al., 2008; Gueorguieva, Sindelar, Wu, & Gallo, 2011; Zheng, 2008). Using an IV approach in the Survey of Health, Ageing and Retirement in Europe (SHARE) data, Godard (2016) also showed that retirement caused an increase in the probability of being obese among men retiring from strenuous jobs, but no significant results were found among women. Using Dutch male data, Nooyens and colleagues (2005) echoed findings that retiring from strenuous jobs can be detrimental for men, although they did not include a group of female retirees against which to compare this result. The only exception is Stenholm and colleagues (2017)’s work based on the Finnish Public Sector study, which showed that retirement was associated with slight weight loss in men retiring from sedentary jobs and a slight weight gain in women retiring from diverse and physically active jobs.

Nine longitudinal studies assessed the associations between retirement and diabetes or hypertension (Supplementary Table S2c), and five of them indicated no association. To elaborate, using the French GAZEL cohort, Westerlund and colleagues (2010) found that the cumulative prevalence of self-reported diabetes increased with age, with no break in the trend around retirement. Oksanen and colleagues (2011) found that purchases of diabetes medication in Finland were not altered by retirement, regardless of whether it was statutory retirement or early retirement. The remaining five longitudinal studies were all based on the HRS in the United States. Three of them did not find any significant effects of retirement on self-reported diabetes or hypertension (Coe & Lindeboom, 2008; Horner & Cullen, 2016; Zheng, 2008). In contrast to these nonsignificant results, Insler (2014) suggested that retirement decreased the onset of diabetes and hypertension, but Dave and colleagues (2008) found that retirement was associated with increased self-reported diabetes, even after limiting the study samples to healthy workers prior to retirement. These five studies focus on the same country and, therefore, the conflicting findings cannot be explained by differences in the institutional setting or culture. The possible explanation is that these studies used different analytic methods. Dave and colleagues (2008) used individual fixed effects to control for time-invariant heterogeneity across individuals, and Insler (2014) used workers’ self-reported probabilities of working past ages 62 and 65 as the IV, whereas others used the earliest entry ages for pension or health insurance scheme as the IVs (Coe & Lindeboom, 2008; Horner & Cullen, 2016; Zheng, 2008). One limitation of the above studies on diabetes and hypertension is that their results may be biased due to the unawareness of disease. Two studies used objectively measured blood pressure. Xue and colleagues (2017) found that retirement in China was accompanied by a lower diastolic blood pressure and a slowdown in the increase of systolic blood pressure over time. Ekerdt, Sparrpw, Glynn, and Bosse (1984) found a significant increase in blood pressure for retirees compared with workers over a 3-year span in the U.S. Normative Aging Study. The retirement-related changes of blood pressure found in these two studies are small (though significant); therefore, they may not be of sufficient magnitude to conclude that retirement had a clinically significant effect on hypertension. Only one longitudinal study used objective biological measures of the metabolic syndrome and this showed that retirement increased the risk of having metabolic syndrome (Behncke, 2012).

In summary, results from the United States and several European countries (except Finland) consistently show that retirement is associated with increased adiposity among those retired from physically demanding jobs. In terms of diagnosed diabetes and hypertension, five out of nine longitudinal studies suggest no effect of retirement.

### Retirement and Physical Activity (PA)

Among the included studies, 36 examined the effects of retirement on PA (Supplementary Table S2d). The absence of work activities provides an opportunity for free time; thus, it is not surprising to see that many studies showed an increase in leisure-time PA after retirement (Brown, Heesch, & Miller, 2009; Eibich, 2015; Henkens, van Solinge, & Gallo, 2008; Holstila, Manty, Rahkonen, Lahelma, & Lahti, 2017; Insler, 2014; Koeneman et al., 2012; Kuvaja-Köllner, Valtonen, Komulainen, Hassinen, & Rauramaa, 2013; Lahti & Laaksonen, 2011; Midanik, Soghikian, Ransom, & Tekawa, 1995; Motegi, Nishimura, & Terada, 2016; Oshio & Kan, 2017; Sjösten et al., 2012; Stenholm et al., 2016; Syse et al., 2017).

While evidence of increased leisure-time PA following retirement was consistently found in the literature, retirees were also more likely to have increased sedentary behavior at the same time (Leskinen et al., 2018). A longitudinal study among French participants showed that retirement was associated with about a 2 hr/week increase in leisure-time PA, especially increased duration of moderate activities and walking, but retirees increased the amount of time they spent watching TV by about 2 hr/week (Touvier & Bertrais, 2010). Similar results showing increases in both leisure-time PA and sedentary behavior were found among Americans (Evenson, Rosamond, Cai, Diez-Roux, & Brancati, 2002), Australians (Sprod et al., 2017), and French people (Menai, Fezeu, & Charreire, 2014).

Although retirement has been repeatedly linked to increasing leisure-time PA, retirement may reduce work- and transport-related PA in turn. When it comes to total PA levels, several studies showed a decreased trend following retirement. For example, Slingerland and colleagues (2007) showed that although Dutch retirees participated in leisure activity more often than employees, this was not enough to compensate for the cessation of work-related transportation activity, leading to a reduction in total PA among retirees. Scottish retirees did too little PA outside work to compensate for the loss of work-based activity, thus less often met the PA recommendations (Berger, Der, Mutrie, & Hannah, 2005). Less walking for transportation was also found among Australian retirees (Turrell, Hewitt, & Haynes, 2014). On the contrary, other studies have suggested that retirement increased total PA (Celidoni & Rebba, 2017; Ding et al., 2016; Feng, Croteau, Kolt, & Astell-Burt, 2016; Kesavayuth, Rosenman, & Zikos, 2018; Mein, Shipley, Hillsdon, Ellison, & Marmot, 2005; Müller & Shaikh, 2018).

Some studies have tested the moderating effect of preretirement characteristics. Barnett, van Sluijs, Ogilvie, and Wareham (2012) found a decline in overall PA among British retirees, but the magnitude of association was larger for men and women who retired from manual social class occupations. Jones, Li, Aiello, O’Rand, and Evenson (2018) found that total PA declined after retirement among individuals of low socioeconomic position but remained stable among those of high socioeconomic position. In this study, socioeconomic position was measured by education, household income, and wealth. An even stronger moderating effect of preretirement occupation was found in Chung and colleagues (2009)’s work in the HRS study, in that total PA decreased with retirement from a physically demanding job, but increased with retirement from a sedentary job. Kämpfen and Maurer’s study (2016), which was also based on the HRS, estimated an increase in compliance with PA guidelines in response to retirement and beneficial effects were larger for better-educated individuals and persons with higher levels of household wealth. On a similar note, several studies from European countries found that a beneficial effect of retirement on PA was evident among those who retired from higher employment grades (Mein et al., 2005; Stenholm et al., 2016) and those with higher education levels (Celidoni & Rebba, 2017).

Two studies suggested that the PA level before retirement may also modify the effect of retirement. Using Europen SHARE data, Kesavayuth and colleagues (2018) found that retirement increased vigorous and moderate PA among those who abstained from exercise at baseline. On the contrary, Aggio and colleagues (2018) used the British Regional Heart Study and found that leaving employment is associated with an increase in PA in men who were already at least occasionally active at baseline. This study did not distinguish unemployment from retirement and only included men; thus, its results may be less comparable with other studies.

Studies on PA and retirement conclude that retirement was associated with increases in both leisure-time PA and sedentary behavior. No clear pattern emerged regarding total PA. Social class seems to moderate the association, in that socially disadvantaged people and individuals retired from physically demanding jobs, experience a less beneficial effect or a bigger adverse effect on total PA in response to retirement, which may reflect differences in initial levels of work-related PA and differences in opportunities to participate in leisure-time PA. The moderating role of baseline PA is less clear.

### Retirement and Smoking Status

In the literature, 14 longitudinal studies tested the association between retirement and smoking (Supplementary Table S2e). Two studies using the U.S. HRS data showed contradictory results, with Insler (2014) suggesting that retirement was linked with a decreased probability of smoking but Ayyagari (2016) showing an increased probability. Again, the conflicting results are most likely due to the different IVs used. A third U.S. longitudinal study using the Kaiser Permanente Retirement Study found no difference between working and retired people in the probability of smoking (Midanik et al., 1995). Müller and Shaikh (2018) used European SHARE data and did not find any significant effect of retirement on smoking. However, longitudinal studies conducted in the United Kingdom (Lang, Rice, Wallace, Guralnik, & Melzer, 2007; Morris et al., 1992) and Germany (Eibich, 2015) showed that those who retired were more likely to quit smoking. Four longitudinal studies assessing the effect of retirement on smoking were conducted outside the United States and Europe. Xue and colleagues (2017) did not find any association between retirement and smoking among Chinese and neither did Motegi et al. (2016) in the Japanese Study of Aging and Retirement (JSTAR). However, using the Longitudinal Survey of Middle-Aged and Older Adults in Japan, Oshio and Kan (2017) found that retirement accelerated the rate of smoking cessation among men only. One Australian study found that retirement was associated with reduced smoking (Ding et al., 2016).

There were two longitudinal studies focused on potential moderators of the association. A Dutch cohort study showed that during the 6-year follow-up, people who retired involuntarily had both a higher risk of increased smoking and a lower risk of decreased smoking, and voluntary retirement was not associated with smoking status (Henkens et al., 2008). Using European SHARE data, Kesavayuth and colleagues (2018) found that retirement led to less smoking for those who smoked before retiring and non-smokers did not increase smoking upon retirement.

In summary, most studies (12 out of 14) have shown that retirement was associated with either decreased smoking or had no effect on smoking.

### Retirement and Alcohol Consumption

Twenty-seven longitudinal studies have assessed the relationship between retirement and drinking behavior or alcohol problems (Supplementary Table S2f). Some retirees may turn to alcohol to fill leisure time and cope with the stresses associated with retirement as a major life change. A number of studies support this idea that retirement increases alcohol consumption and the incidence of drinking problems. One study in Germany showed retirement to decrease the probability of abstaining from alcohol (Eibich, 2015) and two studies based on SHARE suggested that retirement leads to a significant increase in the frequency of alcohol intake (Kesavayuth et al., 2018; Müller & Shaikh, 2018). Also using SHARE, Celidoni and Rebba (2017) found that retirement increased the risk of drinking every day for men (borderline significant) but not for women. Two French studies based on the GAZEL cohort suggested that retirement increased the risk of excessive alcohol consumption, temporarily in most people and permanently in the small group of women managers (Tamers et al., 2014; Zins et al., 2011). Two U.S. studies based on the HRS also found retirement to be associated with increased alcohol consumption, particularly among those with a preretirement history of problem drinking (Perreira & Sloan, 2001; Wang, Steier, & Gallo, 2014). A study based on a 2-year panel data among blue-collar Americans showed that retirement generally heralds no great shift in alcohol consumption or drinking patterns; however, retirees were twice as likely to engage in periodic heavy drinking as those who continued to work (Bacharach, Bamberger, Sonnenstuhl, & Vashdi, 2004).

Other researchers, however, argue that work stress and job-based drinking cultures represent a greater risk to workers’ drinking behavior than retirement does. If this is the case, retirement may provide a chance to eliminate these risk factors from workers’ lives. For example, a study among older people (60–96 years) in Japan suggested that alcohol consumption tends to drop significantly among nonworking people (retired and unemployed combined), and the authors ascribed the use of alcohol as a “social lubricant” within Japanese work culture (Gee et al., 2007). Motegi and colleagues (2016) using the JSTAR found that men reduced their alcohol consumption postretirement more than women and suggested that the most likely reason for this is that men, in general, consume much more alcohol than women, so men have relatively more room to decrease their consumption. Xue and colleagues (2017) found that retirement was accompanied by a reduction in the probability of being a heavy alcohol drinker in the Chinese context as well. Neve, Lemmens, and Drop (2000) conducted an analysis of a 9-year longitudinal study in the Netherlands and found retirement to be associated with a decrease in alcohol consumption and alcohol-related problems. Using the HRS, Bob and colleagues estimated 10-year drinking trajectories and suggested that retirement may decrease alcohol consumption among women only (Bobo & Greek, 2011; Bobo, Greek, Klepinger, & Herting, 2013). While Rodriguez and Chandra (2006), utilizing data from the U.S. National Survey of Families and Households, found that retired men were more likely to reduce drinking but there was no effect for retired women. Syse and colleagues (2017) found that retirement in Norway influenced alcohol intake in both directions, that retirees were more likely to both increase and reduce their alcohol intake. Other studies in Australia (Ding et al., 2016), the United Kingdom (Iparraguirre, 2015; Morris et al., 1992), Japan (Oshio & Kan, 2017), and the United States (Brennan, Schutte, & Moos, 2010; Glynn, De Labry, & Hou, 1988; Platt, Sloan, & Costanzo, 2010) suggested no association between retirement and alcohol consumption or drinking problems.

Some studies focused on potential moderators of the association. In an analysis of blue-collar retirees (Bacharach, Bamberger, Biron, & Horowitz-Rozen, 2008), those who had higher preretirement job satisfaction demonstrated greater alcohol consumption and more problems related to drinking in retirement. The conditions of the workplace exit itself, such as whether the decision is voluntary, may also impact drinking behaviors. For instance, Henkens et al. (2008) explored voluntariness of retirement in the Netherlands and concluded that involuntary retirees may use alcohol to cope with the stress of sudden change in their employment status.

In aggregate, based on the evidence, a clear conclusion cannot be drawn on the effect of retirement on drinking behaviors or alcohol problems. This is probably because of the different study designs applied. For example, the measures of drinking outcomes vary between studies, with some using the risk of problem drinking or periodic heavy drinking, some using the risk of nondrinking, some using drinking trajectories (increased/declined/stable/other), and others using average alcohol consumption. In addition, the study samples were from different countries with different drinking cultural settings, both in and out of work. Preretirement conditions, such as high job satisfaction or workplace stress, may also influence the overall use of and problems with alcohol.

### Retirement and Diet

Changes in food habits after retirement have been investigated in only four longitudinal studies (Supplementary Table S2g). A French study found that transition to retirement was associated with unhealthier dietary intakes such as a decrease in intake of fruits, proteins, and some vitamins as well as an increase in intakes of fatty and sweet products, saturated fatty acids, and sodium. Changes of dietary intakes with retirement were particularly marked in men with the lowest income at baseline (Hassen et al., 2017). Another French longitudinal study reported that the amount of dietary nutrients consumed remained the same before and after retirement (Lauque, Nourashemi, Soleilhavoup, Guyonnet, & Bertiere, 1998). Using the Helsinki Health Study cohort, Helldán and Lallukka (2012) suggested that the transition to retirement led to healthier food habits among women, but a similar trend was not found among men. An Australia study suggested that there was no significant association between retirement and fruit and vegetable consumption (Ding et al., 2016). In summary, no clear pattern in associations between retirement and diet emerged from reviewed studies.

### Retirement and Blood Biomarkers

Only one study, by Behncke (2012), examined the effect of retirement on CVD blood biomarkers (Supplementary Table S2h). The results showed that retired individuals are 7% more likely to have a high fibrinogen concentration. C-reactive protein and hemoglobin were not significantly associated with retirement.

## Discussion

This review showed that retirement impacts CVD and its risk factors in different ways. Included studies either showed increased CVD events among retirees or found no significant association between retirement and CVD. In terms of CVD metabolic risk factors, there is strong evidence that retirement increased adiposity measures among those retired from physically demanding jobs, and more than half of the included studies suggested no effect of retirement on diabetes and hypertension. For behavioral risk factors, this review indicated strong evidence for retirement not increasing smoking, and conflicting evidence for retirement having an effect on drinking and PA. Few studies assessed the effect of retirement on diet and blood biomarkers.

A number of factors may underlie the inconsistency in these research findings. First, inconsistencies may stem from the way in which researchers conceptualize retirement, with some using self-reported employment status, while others use SPA. Other sources of inconsistency may be that the study samples vary with respect to age ranges, gender, country of origin, measures of health outcomes, study designs, and statistical methodologies. Among the sources of inconsistency, the country of the study population seems to play an important role in the results. For example, empirical studies in the United States often found no significant effect of retirement on CVD, while studies in European countries, except France, showed a consistent detrimental effect of retirement on CVD. As suggested by the life-course perspective, individuals’ responses to retirement are often related to features of the institutional environment shaping their choices of retirement (Riley & Riley, 1994). Thus, different retirement policies and social-welfare legislation between the United States and European countries are likely to be one of the factors influencing the health and well-being of individuals after retirement. For instance, there is no statutory retirement age in the United States, while there are statutory retirement ages in European countries, although some European countries have recently outlawed compulsory retirement. In addition, whether people are categorized as unemployed or as retired may depend on the country specific social security system, and unemployment has an adverse effect on health.

It is also important to note that, regardless of heterogeneity, none of the included studies has found any beneficial effects of retirement on CVD, and only a few of them suggest retirement reduced metabolic risk factors of CVD. Except for smoking, the general finding regarding retirement and CVD and CVD risk factors is contrary to the potential relief effect of retirement on mental health found in the literature (van der Heide, van Rijn, Robroek, Burdorf, & Proper, 2013). However, studies that showed negative health effects should be interpreted with caution. Many assessed the health effects of retirement by comparing retired people with employed people. This method may overestimate the detrimental effect of retirement on health since people who stay in the labor market are generally healthier than retirees. There is consistent evidence that people who have CVD, diabetes, or hypertension are more likely to retire (Dwyer & Mitchell, 1999; Pedron, Emmert-Fees, Laxy, & Schwettmann, 2019), and therefore, reverse causality might be most likely occurred when assessing the relationship between retirement and CVD or metabolic risk factors. Some of the longitudinal analytic methods used in some of the studies reviewed might be useful in terms of reducing reverse causality. For instance, the study by Behncke (2012) applied two statistical methods: propensity score matching and IV analysis. Propensity score matching is a useful technique to control for selection biases in observational studies, but this method requires a large enough sample size to process matching, and in the case of retirement, detailed information of the job characteristics and the eligibility for pension and health insurance is needed. The second method employed is using the SPA as an IV. This method controls for confounding using an IV related to the exposure but not to an individual’s background characteristics, therefore is better able to estimate causal effects of the exposure on outcomes. However, its results only apply to the subsample who retired induced by the IV (i.e., SPA here). Some studies (i.e., Gueorguieva et al., 2011; Oksanen et al., 2011; Westerlund et al., 2010; Xue et al., 2017) limited their study samples to retirees whose health outcomes had been measured repeatedly both before and after retirement and compared the trajectories of outcomes of interest before and after retirement among retirees. This piecewise method largely reduced the probability of reserve causality, although did not totally remove this problem.

Another important point regarding studies of retirement is distinguishing between the effect of retirement and the effect of aging. To control for the effect of aging, all included studies have adjusted for age. However, more advanced methods are needed to provide reliable evidence that the effect of retirement is truly distinct from the effects of aging. Studies (i.e., Gueorguieva et al., 2011; Oksanen et al., 2011; Westerlund et al., 2010; Xue et al., 2017) used interrupted time-series before and after retirement which could better control the effect of aging.

Another common limitation of reviewed studies is the inappropriate handling of missing data. Most studies used complete case analysis, and only a few studies had multiply imputed missing data in the analysis (Xue et al., 2017; Forman-Hoffman et al., 2008; Henkens et al., 2008; Gee et al., 2007; Iparraguirre, 2015; Rodriguez and Chandra, 2006). Use of complete case analysis could lead to biased estimations and reduce the statistical power. Finally, most studies were conducted in the United States and European countries and very few were conducted among less developed countries which might keep this review from providing a comprehensive overview of how retirement impacts CVD in low-income countries.

### Future Perspectives

Going forward, it will be valuable to further explore the mechanisms potentially linking retirement and CVD. There is only one study (Méjean et al., 2013) which tested potential mediators and found that dietary factors and other health behaviors explained 15% and 30% of the increased risk for CVD in retirees, respectively. Except for health behavioral factors, it is unclear whether the changes in mental and physical health associated with retirement also influence the development of CVD. Additionally, it is important to understand whether the mechanisms differ between countries, which will help to better understand why studies in the United States often find no significant effect of retirement on CVD, while most studies in European countries show a detrimental effect of retirement on CVD. More research on the effect of retirement on blood biomarkers is needed, as blood biomarkers are collected by nurses; thus, they are not affected by self-reporting bias and have the ability to identify high-risk individuals long before the development of CVD events. Finally, understanding factors which may interact with individuals’ adaptation to retirement is particularly important and future research could focus on factors such as gender, occupational social class, and workplace stress.

### Policy Implications

Findings from this systemic review have shown the complex nature of the underlying mechanism through which retirement has effects on CVD and CVD risk factors. Individuals’ responses to retirement are often related to their life course experience and the features of the institutional policy environment shaping their timing and nature of retirement. Rather than simply mirror other countries, policy concerning extending the retirement age needs to focus on the diverse nature of transitions into retirement and on ensuring they are suited to the individual.

## Funding

J.H. was partially supported by the Economic and Social Research Council (ES/L0028921/1). A.M. was partially supported by the Economic and Social Research Council (ES/R008930/1).

## Conflict of Interest

None reported.

## Supplementary Material

gnz062_suppl_Supplementary_Material
