# Supplementary material for: The Impact of Retirement on Cardiovascular Disease and Its Risk Factors: A Systematic Review of Longitudinal Studies
Source: Gerontologist. 2019 May 15;60(5):e367–77. doi: 10.1093/geront/gnz062 (PMC7362617; doi:10.1093/geront/gnz062)
Supplement: gnz062_suppl_Supplementary_Material [file gnz062_suppl_supplementary_material.docx]

**Supplementary material**

**Table 1s.a** Searching commands in Medline, Embase, PsycINFO, and Social Policy and Practice. ^^[[1]](#footnote-1)^^

1. exp Retirement/

2. retirement.tw.

3. retired.tw.

4. 1 or 2 or 3

5. (cardiovascular adj disease$).tw.

6. exp Cardiovascular Diseases/

7. (heart adj disease$).tw.

8. (heart adj attack).tw.

9. (coronary adj3 disease$).tw.

10. angina.tw.

11. (myocardial adj infarction).tw.

12. (isch?mi$ adj3 heart).tw.

13. (heart adj failure).tw.

14. (heart adj3 infarc$).tw.

15. (cardiac$ adj3 disease$).tw.

16. or/5-15

17. (cerebrovascular$ or (cerebral adj vascular)).tw.

18. (infarct$ or insch?emi$ or thrombo$ or emboli$ or apoplexy).tw.

19. 17 and 18

20. (stroke$ or poststroke$ or cva$).tw.

21. (hemipleg$ or hemipar$ or post-stroke).tw.

22. (brain adj attack).tw.

23. (cardiovascular adj3 (outcome$ or morbidity or event$)).mp.

24. exp Hypertension/

25. (blood adj pressure).tw. Insert Search Statement

26. exp Metabolic Diseases/ Insert Search Statement

27. ((metabolic adj syndrome) or (metabolic adj factor$)).tw.

28. hyperlipid$.tw.

29. hypercholesterol$.tw.

30. cholesterol$.tw.

31. dyslipidaemia.tw.

32. exp lipid blood level/

33. triglycerides.tw.

34. (blood adj fat$).tw.

35. (HDL or LDL).tw.

36. exp Hemoglobin A, Glycosylated/

37. HbA1c.tw.

38. inflammation.tw.

39. (inflammatory adj markers).tw.

40. exp Diabetes Mellitus/

41. diabetes.tw.

42. exp Body mass index/

43. BMI.tw.

44. exp overweight/ or exp obesity/

45. ((physical adj3 inactiv$) or (physical adj activity) or (sport$ adj3 participat$)).tw.

46. exp Sedentary Lifestyle/ or Exercise/

47. exp Smoking/

48. exp Tobacco/

49. tobacco$.tw.

50. cigarette$.tw.

51. exp Alcohol Drinking/

52. drinking.tw.

53. alcohol$.tw.

54. exp Diet/

55. exp Food Habits/

56. eating.tw.

57. or/19-56

58. 16 or 57

59. 4 and 58

60. remove duplicates from 59

**Table 1s.b** Searching commands in Social Science Citation Index^^[[2]](#footnote-2)^^.

1. TS=retirement

2. TS=(retired or retire*)

3. #2 OR #1

4. TS=(cardiovascular NEAR/1 disease$)

5. TS=cardiovascular disease$

6. TS= (heart NEAR/1 disease$)

7. TS=(coronary NEAR/3 disease$)

8. TS=angina

9. TS= (heart NEAR/3 attack)

10. TS= (myocardial NEAR/1 infarction)

11. TS=(isch*mi$ NEAR/3 heart)

12. TS= (heart NEAR/1 failure)

13. TS= (heart NEAR/3 infarc*)

14. TS=(cardiac NEAR/3 disease$)

15. #14 OR #13 OR #12 OR #11 OR #10 OR #9 OR #8 OR #7 OR #6 OR #5 OR #4

16. TS= (cerebrovascular or (cerebral NEAR/1 vascular))

17. TS=(infarct* or insch*emi* or thrombo* or emboli* or apoplexy)

18. #17 AND #16

19. TS= (stroke$ or poststroke$ or cva$)

20. TS=(hemipleg* or hemipar* or post-stroke)

21. TS= (brain NEAR/3 attack）

22. TS= (cardiovascular NEAR/3 (outcome$ or morbidity or event$))

23. TS=Hypertension

24. TS= blood pressure

25. TS= Metabolic Disease$

26. TS= ((metabolic NEAR/1 syndrome) or (metabolic NEAR/1 factor$))

27. TS= hypercholesterol$

28. TS=cholesterol$

29. TS=dyslipidaemia

30. TS=lipid blood level

31. TS= triglycerides

32. TS= (blood NEAR/1 fat$)

33. TS= (HDL or LDL)

34. TS= glycosylated haemoglobin

35. TS= HbA1c

36. TS=inflammation

37. TS = (inflammatory NEAR/1 markers)

38. TS= diabetes

39. TS= body mass index

40. TS=BMI

41. TS= (overweight or obesity)

42. TS= ((physical NEAR/3 inactiv*) or (physical NEAR/1 activity) or (sport* NEAR/3 participat*))

43. TS= (Sedentary Lifestyle or Exercise)

44. TS= (smoking or tobacco$ or cigarette$)

45. TS= (alcohol$ or drinking)

46. TS= (diet or eating or food habits)

47. #46 OR #45 OR #44 OR #43 OR #42 OR #41 OR #40 OR #39 OR #38 OR #37 OR #36 OR #35 OR #34 OR #33 OR #32 OR #31 OR #30 OR #29 OR #28 OR #27 OR #26 OR #25 OR #24 OR #23 OR #22 OR #21 OR #20 OR #19 OR #18 OR #15

48. #47 AND #3

**Table 2s.a** Longitudinal studies on retirement and CVD

| **Author (year)** | **Data used** | **Sample size, age (mean), and % women** | **Type of analysis & covariates** | **Effects on outcome measure(s)** | **Effect type** | **Overview of findings** |
| --- | --- | --- | --- | --- | --- | --- |
| Kang and Kim (2014) | Korean Longitudinal Study of Aging (2006, 2008, 2010, and 2012) | 10,254  ≥45 y  35.3% | Cox proportional hazard regression analysis: adjusted for age, gender, history of diabetes and hypertension, diabetes, and smoking, heavy drinking, regular exercise, BMI, and income. | **Diagnosed stroke or CVD**  Men and women  Voluntary:  2.595 (1.595,4.074)*  Involuntary:  2.955 (1.908,4.577)*  Men  Voluntary:  2.879 (1.533, 5.409)*  Involuntary:  3.560 (2.055, 6.168)*  Women  Voluntary:  2.410 (1.177, 4.934)*  Involuntary:  1.864 (0.839,4.140) | HR (95%CI) for retired people vs. working people | Both voluntary and involuntary retirement were associated with increase CVD or stroke, and the relationship was stronger among male subjects. |
| Olesen et al.  (2014) | A registry-based cohort study including  all Danish citizens born between 1932 and 1948. | 617,511  60-67 y  44% | Cox proportional hazard regression analysis: adjusted for baseline year, sex, occupational position, education, income, residential area, cohabitation and being immigrant. | **Diagnosed MI**  Men  Newly retired (≤26 weeks):  1.14 (1.05, 1.24)*  Retired workers:  1.10 (1.05, 1.16)*  Women  Newly retired:  0.84 (0.68, 1.02)  Retired workers:  1.14 (1.03,1.27)* | HR (95%CI) for retired people vs. working people | Retirement was associated with a modestly increased risk of MI. |
| Méjean et al. (2013) | Dutch segment of the European Investigation into Cancer and Nutrition study (1993-1997) | 33,106  20-70 y  94.9% of retirees and 66.5% of working people  are women | Cox proportional hazard regression analysis: adjusted for gender and age | **Diagnosed CVD** HR:1.20 (1.05, 1.37)*  **Diagnosed stroke** HR: 1.05 (0.85,1.31)  **+ dietary factors:**  15.15% (9.56,24.04)  **+ all lifestyle factors:**  30.30% (22.07,38.05) | HR (95%CI) for retired people vs. working people  %: percentage reduction in HR of CVD | Retired subjects more often suffered from CVD than working people, but not from stroke.  Dietary factors and lifestyle factors explained 15% and 30% of the increased HRs for CVD in the retired group, respectively. |
| Behncke (2012) | English Longitudinal Study of Ageing (2002-2006) | 1,439  >=50 y  50.5% | 1. State retirement age (65 years) as an IV for retirement: adjusted for age, gender, job characteristics, anticipation to stay in work, and pre-retirement health  2. Propensity score matching | **Diagnosed CVD (angina, MI or stroke)**  IV: 0.074  Matching: 0.039* | Coefficients from non-parametric IV and non-parametric matching methods. | Retirement people had significantly higher CVD incidence than working people. |
| Dupre et al. (2012) | US Health and Retirement Study (1992-2010) | 13,451  51-75 y (55.2 y)  48.8% | Cox proportional hazard regression analysis: adjusted for age, gender, race/ethnicity, marital status, geographic region, educational household income, health insurance, current smoking status, alcohol use, vigorous physical exercise, regular cholesterol screenings, BMI, hypertension, diabetes, ADLs, and number of depressive symptoms. | **Diagnosed AMI**  1.20 (0.971.48) | HR (95%CI) for retired people vs. working people | Retirement was not associated with AMI incidence. |
| Masoudkabir, (2012) | Isfahan cohort study (three counties in central Iran) (2001, 2003, 2005/06) | 6,504  35-75 y (50.7 y)  48.1% | Cox proportional hazard regression analysis: adjusted for gender, age, baseline smoking status, BMI, and hypertension. | **Diagnosed CVD (ischemic heart disease and stroke)**  Manual worker:  0.84 (0.29,2.39)  Non-manual worker:  0.86 (0.30,2.44)  Retired people:  0.65 (0.22,1.86) | HR (95%CI) (housewife as reference) | There was no significant occupational difference on CVD incidence. |
| Moon (2012) | US Health and Retirement Study (1998-2008) | 5,422  >=50 y  54% | Discrete-time survival analyses based on pooled logistic regression: adjusted for age, sex, childhood and adult SES, behaviour, and co-morbidities. | **Diagnosed CVD (stroke or MI)**  First year after retirement:  1.55 (1.03, 2.33)*  ≥2 years after retirement  1.35 (0.96, 1.91) | HR (95%CI) (fully retired people vs. full time working people) | CVD risk was increased after retirement, especially in the first year. |
| Westerlund, (2010) | France, GAZEL study (1989-2007) | 14,104  20.3% | Multivariate: adjusted for sex, year of birth, and marital status. | **Cumulative prevalence of diagnosed CVD** **(angina, MI or stroke)** | Not stated | Cumulative prevalence of CVD increased with age, with no break in the trend around retirement. |
| Bamia (2008) | Greek segment of the European Investigation into Cancer and Nutrition study (1994-2006) | 16,827  20-86 y  47% | Cox proportional hazard regression analysis: adjusted for age at baseline, education, smoking, PA, BMI, total energy intake and alcohol intake. | **Death from circulatory diseases**  1.73 (1.10, 2.73)* | HR (95%CI) for retired people vs. working people | Retired people have higher risk of death from circulatory disease than working people. |
| Coe and Lindeboom  (2008) | US Health and Retirement Study (1992-2005) | 3,657  50-70 y (59.19 y)  0% | Offered a  retirement window as an IV for retirement: adjusted for white/blue-collar worker, age, age^2^, education, marital status, net worth deciles, race, Hispanic, US-born, the number of children, and wave. | **Diagnosed MI**  2 years: -0.0421  4 years: 0.0779 | Coefficients from dynamic IV analysis. | Retirement had no effect on MI in the short run (within 2 years) or long run (within 4 years). |
| Dave et al. (2008) | US Health and Retirement Study (1992-2005) | 4,951 to 5,289 50-75 y | Fixed effect: adjusted for gender, ethnicity, education, marriage, religious, income, health insurance, parents’ age and education, native-born, risk averse, planning horizon, fixed effects of age, year, census division, and individual. | **Diagnosed heart disease and stroke**  Heart: 0.0268***  Stroke: 0.0173***  Restricted to samples who are healthy pre-retirement  Heart: 0.0148*  Stroke: 0.0075*  Restricted to samples who are healthy pre-retirement &consistently insured  Heart: 0.0084  Stroke: 0.0052 | Marginal effect of complete retirement on health | Retirement was associated with increased incidence of heart disease and stroke, but not once samples were restricted to those consistently insured in all waves. |
| Petrelli et al. (2006) | Turin Longitudinal Study in Italy (1997-2002) | 523,755  35-74 y  52.1% | Hierarchical Poisson models: adjusted for age, education, income and area of birth. | **Diagnosed CVD**  Men:  1.14 (1.06-1.23)* Women:  1.20 (1.03-1.41)*  **Death from CVD** Men:  1.54 (1.26-1.89)*  Women:  2.06 (1.43-2.97)* | Coefficients for retired people vs. working people | Retired people have higher incidence of CVD and higher risk of death from CVD than working people. |
| Morris et al. (1994) | British Regional Heart Study (1978/80-1983/85) | 6,191  40-59 y (49.9 y)  0% | Cox proportional hazard regression analysis: adjusted for age, town, social class, smoking, alcohol intake, and pre-existing disease at initial screening. | **Death from circulatory disease**  1.81 (1.12, 2.93)* | HR (95%CI) for retired people vs. working people | Retired people have higher risk of death from circulatory disease than working people. |
| Vallery-Masson (1981) | Parisian male managers (1976-1979), France. | 156  63-64 y  0% | Adjusted for age and baseline income | **Diagnosed CVD incidence**  Retired: 27%  Working 16% | Percentage of people have diagnosed CVD in the follow-up | Retired men did not show significantly higher CVD incidence. |

HR: hazard ratio. OR: odds ratio. *p<0.05; **p<0.01; ***p<0.001

**Table 2s.b** Longitudinal studies on retirement and adiposity measures

| **Author (year)** | **Data used** | **Sample size, age (mean), and % women** | **Type of analysis & covariates** | **Effects on outcome measure(s)** | **Effect type** | **Overview of findings** |
| --- | --- | --- | --- | --- | --- | --- |
| Nishimura et al. (2018) | US Health and Retirement Study (1996-2011), English  Longitudinal Study of Ageing Survey of Health (2002-2014),  Ageing, and Retirement in Europe (2004-2012), Japanese Study of Ageing and Retirement (2007-2013), Korean Longitudinal Study of Aging (2006-2012) | 50 y+  HRS: 20,645  ELSA: 8,230  SHARE: 54,110  JSTAR: 2,222  KloSA: 7,458 | Retirement benefit eligibility ages as IVs for retirement: adjusted for age, age squared, married, number of children, household income, housing, household total wealth, region and wave. | **BMI**  US:1.406***  Japan:2.796***  England:0.179  Demark:0.121  France: -0.056  Germany: -0.331  Switzerland:0.776  Korea:0.532 | Fixed effects IV coefficients | BMI increased after retirement in the US and Japan, but retirement had no effect in England, Demark, France, Germany, Switzerland, and Korea. |
| Syse et al. (2017) | Norwegian  study on Life course, Ageing and Generation  (2002,2007) | 546  57-66 y (60.5 y)  47% | Multinomial  logistic regression: adjusted for age, gender, marital status, education,  public versus private sector employment, number of hours worked  per week,  stressful work situations, and sickness absence over the last 12 months | **Weight loss**  1.75 (1.12, 2.71)  **Weight gain**  0.78 (0.47, 1.31) | OR  for retired people vs. working people (95%CI) | Retirees were more likely to report an increase in  weight loss. |
| Stenholm et al. (2017) | Finnish Public Sector study  (2000-2013) | 5,426  79.4% | Linear regression analyses with generalized estimation equations: adjusted for retirement age, SES, time-varying PA, alcohol consumption and smoking as well as marital status, number of chronic diseases, job strain and BMI before retirement | **Weight**  men retiring from sedentary jobs: −0.18 (−0.30, −0.05)  women retiring from diverse jobs:  0.14 (0.08,0.20)  women retiring from physically heavy jobs: 0.31 (0.16, 0.45) | Coefficients (95%CI) | Retirement is associated with slight weight loss in men retiring from sedentary jobs and a slight weight gain in women retiring from diverse and physically heavy jobs |
| Xue et al. (2017) | China Health and Nutrition Survey (1991–2011) | 1,084 (46.4 y)  (41.3%) | Piecewise regression: adjusted for  sex, education, province, spouse's working status/no spouse in the household, occupational skill level, PA level at the workplace, and per capita annual household income, smoking, drinking, and age at retirement  age in 1991 | **Change of BMI:**  Linear slope change  0.011 (−0.055, 0.077)  Quadratic slope change 0.001 (−0.002, 0.004)  **Change of waist circumference**  −0.142*** (−0.249, −0.035) | Coefficients of piecewise regression | Retirement was accompanied by a reduction in diastolic blood pressure, a slowdown in the increase of both systolic blood pressure and waist circumference. No association with BMI was found. |
| Godard (2016) | Survey of Health, Ageing and Retirement in Europe (2004, 2006 and 2010-2011) | 2,599  50-69 y  (59.8 y)  54.7% | Early retirement age as an IV for retirement: adjusted for age, age^2^, wave, and live with spouse. | **BMI:**  Men: 0.419  Women: 0.733  **Probability of being overweight/obese (25≤BMI):**  Men: 0.050  Women: 0.085  **Probability of being obese** **(BMI ≥30):**  Men: 0.115*  Women: 0.026  Men (strenuous job): 0.104*  Women (strenuous job): -0.037 | Fixed effects IV coefficients | Retirement induced by early retirement rules causes a 11.5% increase in the probability of being obese among men within 2-4 years, which is driven by men retiring from strenuous jobs and by those who were already at risk of obesity. No significant results are found among women. |
| Eibich (2015) | German Socio-Economic Panel Study (1984-2004) | More than 20,000 individuals  55-70 y  (61.7 y)  51.8% | Regression Discontinuity Design using eligibility ages (60 and 65) as exogenous variation in retirement: adjusted for age, gender, lived in East Germany in 1989, education, occupation physical and mental strain, and partner is retired. | **BMI:** -0.424 | Coefficient | No significant impact of retirement on BMI was found. |
| Monsivais et al. (2015) | European Prospective Investigation of Cancer Norfolk study in the UK (1993-97) | 7,201  39-76 y  52.5% | Multivariate: adjusted for age, sex, education, smoking, and measured body weight at baseline. | **Body weight change over follow-up years:**  0.49 (0.42, 0.57) | Annualised mean change (95% CI), kg/year | Weight changes associated with retirement were similar to those staying in work, so retirement was not associated with body weight change. |
| Gueorguieva, (2011) | US Health and Retirement Survey (1992- 2002) | 2,096  (56.7 y)  42.8% | Multivariate: adjusted for year before retirement, gender, race, education, marriage, health behaviours, economic status, job characters, non-housing wealth, health insurance, and attrition/dropout. | **BMI trajectories**  **Service:** 0.08*  **Other blue-collar:** 0.09** | Post-retirement slope | Those in service and other blue-collar occupations had significant increases in the slopes of their BMI trajectories after retirement, whereas individuals in white-collar occupations exhibit no change. |
| Touvier (2010) | SU.VI.MAX study in France (1998-2001) | 1,389  45-64 y (Women:  52.3 y, Men: 57.1 y)  49.7% | Multivariate: adjusted for age, education level and baseline value of the corresponding variable. | **Change of BMI in the follow-up**  Retired men: 0.4  Working men: 0.4  Retired women: 0.6  Working women:0.6  **Change of WC in the follow-up**  Retired men: 1.1  Working men: 1.6  Retired women: 1.9  Working women:3.3 | Mean | No difference between retirees and employees in the change of BMI and waist circumference with time. |
| Chung et al. (2009) | US Health and Retirement Study (1992-2002) | 10,565  50-71 y  (60.6 y)  49.4% | 1-year lagged eligibility for the early Social Security benefit, which is 63y, as an IV for retirement: adjusted for age, age2, income, smoking, PA, and spousal factors | **BMI**  Total: 0.242*  Physically demanding job: 0.478*  Sedentary job: 0.043  Initially BMI≥25: 0.294*  Initially BMI<25: 0.073 | Marginal effects of retirement from fixed effects IV | Weight gain with retirement was found among people who were already overweight and those retiring from physically demanding occupations. |
| Zheng (2008) | US Health and Retirement Study (1992-2004) | 6,935  50-73 y  43.2% | Ages of Social Security (62y) and Medicare (65y) eligibility as IVs for retirement: adjusted for age, age^2^, education, ethnicity, income, wealth, number of IADL/ADL limitations, self-rated health, ever being diagnosed cancer, diabetes, heart disease, hypertension, lung disease and stroke. | **BMI**  Men: -0.180  Women: 0.446  Men from strenuous occupation:0.621***  Women from strenuous occupation: 0.091 | Fixed effects IV coefficients | Retirement was associated with higher BMI among men retiring from strenuous jobs.  No significant results were found among women. |
| Forman-Hoffman et al.  (2008) | US Health and Retirement Study (1994-2002) | 3,725  53-63 y  47.2% | Multivariate: adjusted for baseline functional limitations, medical condition, depression, weight, marriage, PA, age, smoking, ethnicity, education, occupation, alcohol consumption and time period. | **>=5% increase in BMI:**  Blue collar women: 1.58 (1.13,2.21)*  White collar women:  1.13 (0.92,1.39)  Blue collar men:  1.05 (0.83,1.34)  White collar men:  0.85 (0.65,1.10) | OR retired vs. working (95%CI) | Retirement was associated with a significant weight gain only among women in blue-collar jobs, but no effect was found among men. |
| Nooyens et al. (2005) | Doetinchem Cohort Study in the Netherlands, (1994/1997-1999/2002) | 288  55-65 y  0% | Adjusted for age | **Weight**  Sedentary job: 0.08  Active job: 0.42*  **Waist circumference:**  Sedentary job: 0.23  Active job: 0.77* | Mean change in the outcomes of retirees in the follow-up | Retired men gained more weight than continued employed men and that the weight gain was greater among retirees from physically active jobs than retirees from sedentary jobs. |
| Morris et al. (1992) | British Regional Heart Study (baseline: 1978-1980, follow up until 1983-1985) | 6,057  40-59 y  0% | Multivariate: adjusted for age, social class and town of residence. | **Loss BMI>10%**  Continuously employed: 2.1  Non-continuously employed: 2.9  **Gain BMI>10%**  Continuously employed: 5.0  Non-continuously employed: 7.5 | % of people who loss or gain BMI | Non-continuously employed men were more likely to either gain or lose weight, whereas weight was stable among the continuously employed men. |

OR: odds ratio. *p<0.05; **p<0.01; ***p<0.001

**Table 2s.c** Longitudinal studies on retirement and metabolic chronic conditions (except for obesity).

| **Author (year)** | **Data used** | **Sample size, age (mean), and % women** | **Type of analysis & covariates** | **Effects on outcome measure(s)** | **Effect type** | **Overview of findings** |
| --- | --- | --- | --- | --- | --- | --- |
| Xue et al. (2017) | China Health and Nutrition Survey (1991–2011) | 1,084 (46.4 y)  (41.3%) | Piecewise regression: adjusted for  sex, education, province, spouse's working status/no spouse in the household, occupational skill level, PA level at the workplace, and per capita annual household income, BMI, smoking, drinking, and age at retirement  age in 1991 | **Change of SBP:**  −0.463 (−0.663, −0.264) *******  **Change of DBP:**  −0.557 ( −0.682, −0.433) *** | Slope change (95%CI) | Retirement was accompanied by a reduction in diastolic blood pressure, a slowdown in the increase of systolic blood pressure. |
| Horner and Cullen (2016) | Secondary data on a cohort of male manufacturing workers in a US setting (1997-2009) | 1,008  55-70 y  0% | Reach age 62 (earliest age received a private pension) as an IV for retirement: adjusted for plant and age polynomial. | **Probability of having hypertension:** −0.00174  **Probability of having diabetes:** 0.0613 | IV coefficients | Retirement induced by private pension age at 62 has no effects on hypertension or diabetes. |
| Insler (2014) | US Health and Retirement Study (1992-2010) | 6,276  ≥50 y  (workers: 59.4; retirees: 66.4)  Workers:43%  Retirees:46% | Workers' self-reported probabilities of working past ages 62 and 65 as an IV for retirement,  adjusted for age, age^2^, gender, black, Hispanic, married, assets, debt, and education. | **Hypertension**  -0.0906***  **Diabetes**  -0.0966*** | Coefficients of IV analysis | Retirement exerts a beneficial  influence on prevention of diabetes and hypertension |
| Behncke (2012) | English Longitudinal Study of Ageing (2002-2006) | 1,439  >=50 y  50.5% | 1. State retirement age (65 years) as an IV for retirement: adjusted for age, gender, job characteristics, anticipation to stay in work, pre-retirement health  2. Propensity score matching | **Diagnosed metabolic syndrome**  matching: 0.072^ꝉ^  IV: 0.069^ꝉ^ | Coefficients from non-parametric IV and non-parametric matching methods. | Retirement people had higher metabolic syndrome incidence than working people, but only at the 10% significance level. |
| Oksanen et al. (2011) | Finnish Public Sector Study cohort (1995-2004) | 11,019  23-67 y  75% | Adjusted retirement age and calendar year | **Diabetes medication purchases**  Not state | Not state | The trend of purchases of diabetes medication was not altered by retirement; no matter it was statutory retirement or early retirement. |
| Westerlund et al. (2010) | France, GAZEL study (1989-2007) | 14,104  20.3% | Multivariate: adjusted for sex, year of birth, and marital status. | **Cumulative prevalence of diagnosed diabetes**  Not stated | Not stated | Cumulative prevalence of CVD increased with age, with no break in the trend around retirement. |
| Coe and Lindeboom  (2008) | US Health and Retirement Study (1992-2005) | 3,657  50-70 y  (59.19 y)  0% | Offered a  retirement window as an IV for retirement: adjusted for white/blue-collar worker, age, age^2^, education, marital status, net worth deciles, race, Hispanic, US-born, the number of children, and wave. | **Diagnosed diabetes**  2 years: 0.0873  4 years: 0.0753  **Diagnosed hypertension**  2 years: -0.0061  4 years: -0.0173 | Coefficients from dynamic IV analysis. | Retirement had no effect on the incidence of diabetes or hypertension in the short run (within 2 years) or long run (within 4 years). |
| Zheng (2008) | US Health and Retirement Study (1992-2004) | 6,935  50-73 y  43.2% | Ages of Social Security (62y) and Medicare (65y) eligibility as IVs for retirement: adjusted for age, age^2^, education, ethnicity, income, wealth, number of IADL/ADL limitations, self-rated health, ever being diagnosed cancer, diabetes, heart disease, hypertension, lung disease, and stroke. | **Ever diagnosed with diabetes**  Retired: -0.008  Retired×strenuous occupation:0.028 | Fixed effects IV coefficients | Retirement has no casual effect on diabetes |
| Dave et al. (2008) | US Health and Retirement Study (1992-2005) | 4,951 to 5,289 50-75 y | Gender, ethnicity, education, marriage, religious, income, health insurance, parents’ age and education, native-born, risk averse, planning horizon, fixed effects of age, year, census division, and individual. | **Diagnosed diabetes**  0.0126***  Restricted to samples who are healthy pre-retirement  0.0126*  Restricted to samples who are healthy pre-retirement &consistently insured  0.0142** | Marginal effect of complete retirement on health | Retirement was associated with increased incidence of heart disease and stroke, but not once samples were restricted to those consistently insured in all waves. |
| Ekerdt et al. (1984) | US Normative Aging Study (1972-1981) | 262  55-70y (63.6 y)  0% | Adjusted for baseline level of the dependent  variable, time between examinations,  age, baseline BMI and  change in BMI | **Change of SBP**  3.44 mmHg*  **Change of DBP**  1.62 mmHg*  **Change of total blood cholesterol**  5.560 mg/dl | Retired vs. working people | Retirement was associated with increased SBP and DBP but not total cholesterol. |

OR: odds ratio. ^ꝉ^ p<0.1; *p<0.05; **p<0.01; ***p<0.001

**Table 2s.d** Longitudinal studies on retirement and PA**.**

| **Author (year)** | **Data used** | **Sample size, age (mean), and % women** | **Type of analysis & covariates** | **Effects on outcome measure(s)** | **Effect type** | **Overview of findings** |
| --- | --- | --- | --- | --- | --- | --- |
| Kesavayuth et al. (2018) | Survey of Health Aging and Retirement in Europe (2004-2013; waves 1, 2, 4, 5) | 5,319  50-75y  48.7% | Eligibility  ages for early and normal retirement as IVs for retirement: adjusted for age, gender, household income, education, household size, marital status,  working in a physically  demanding job,  working in a stressful  job, European region and wave. | **Probability of vigorous activities**  Retired: 0.569***  **Probability of moderate activities**  Retired: 0.572** | Fixed effects IV coefficients | Retirement increased PA |
| Müller and Shaikh (2018) | Survey of Health Aging and Retirement in Europe (2004-2013; waves 1, 2, 4, 5) | 23,589  50 y+ (63.35 y) | Age crossing the official retirement age and the interaction of that indicator with age as IVs | **Probability of vigorous activities**  0.06**  **Probability of moderate activities**  0.06*** | Fuzzy regression discontinuity | Retirement increased physical activities |
| Jones et al. (2018) | US Multi-Ethnic Study of Atherosclerosis (2000-2012) | 4091  45-84 y (57.3 y) 56% | Fixed effect mode. Age as time scale  Adjusted for self-reported health, partnership status, and chronic conditions | **Percentage differences of PA**  Low SEP: 0.76**  High SEP: 1.01 | Coefficient differences | Total PA declined after retirement among individuals of low SEP but remained stable among those of high SEP |
| Aggio et al. (2018) | British Regional Heart Study (1978-2000) | 4,952  49.1+/-5.6 y  0% | Adjusted for number of CVD diagnoses, occupational class, marital status, number of children, region, BMI, arthritis, bronchitis, blood pressure, breathlessness, chest pain, smoking status, alcohol consumption and breakfast consumption | **Probability of trajectories of PA**  low decreasing group=−0.306*** light stable=0.324 ***  moderate increasing groups= 0.847*** | Coefficients | Leaving employment was associated with a decrease in PA in the low decreasing group (β −0.306, p < 0.001) but an increase in the light stable (β 0.324, p < 0.001) and moderate increasing groups (β 0.847, p < 0.001). |
| Leskinen et al. (2018) | Finnish Retirement and Aging Study (2013-2017) | 2,011 (63.2 y)  83% | Linear  regression analyses with generalized estimating equations | **Change of total sedentary time**  1.23 h | Mean change | Total sedentary time increased during the  retirement transition. |
| Sprod et al. (2017) | Multimedia Activity Recall for Children and Adults (2012/13-2013/14) | 124 | Random effects mixed modelling, adjusted for sex, baseline value of dependent variable. | **Change of time spent on**  Work: −122 min ***  Transport: −26 min ***  Chores: +55 min ***  Screen time: +32 min ***  Sleep: +32 min ***  Quiet time: +17 min **  Self-care: +8 min **  PA: +7 min ** | Mean change | Less time was spent in work and transport. There were significant increases in time spent on chores, screen time, sleep, quiet time, self-care and PA. |
| Celidoni and Rebba  (2017) | Survey of Health Aging and Retirement in Europe (2004-2012; waves 1, 2, 4) | 13,464  45-85 y  (65.05 y)  46.3% | Changes in eligibility rules for early retirement and old-age pension across several European countries and over time as IVs for retirement: adjusted for gender, education, age, age2/100, marital status, wealth, number of grandchildren, country, and wave | **Probability of no activities**  -0.042***  White collar:  -0.036**  Blue collar: -0.026  **Probability of no vigorous activities**  -0.084***  White collar:  -0.087**  Blue collar: -0.033  Men: -0.078  Women: -0.087**  Lower education (ISCED 0-4): -0.0571  Higher education (ISCED 5-6): -0.135** | Fixed effects two-stage least squares IV coefficients | The probability of not practicing any PA decreases significantly after retirement, and this effect is stronger for individuals with higher education. |
| Holstila et al. (2017) | Helsinki Health Study cohort (2000-2012, phase 1-3) | 2,902  50+ y (54.4 y)  79% | Generalized estimating equations: adjusted for gender, age, socioeconomic position during phase 1 and time-variant marital status, smoking, limiting long-standing illness and BMI | **Change in leisure-time PA**  Individuals retired during first period:1.10 (1.04–1.17)  Individuals retired during second period:1.10 (1.04–1.16) | Incidence rate ratios (95% CI) | The transition to statutory retirement was associated with an immediate increase in leisure-time PA, which nevertheless diminished during post-retirement years. |
| Oshio and Kan (2017) | The Longitudinal Survey of Middle-Aged and Older Adults in Japan (2005-2014) | 9,283  50-59 y  52.2% | Eligibility for public pension and Eligibility age as IVs: adjusted for living along, have a spouse, provide informal care for family member and wave | **Leisure-time PA:**  Immediate impact of retirement:  Men: 0.475***  Women: 0.283***  Change in the rate of change after retirement:  Men: -0.138**  Women: 0.07 | Fixed effect IV coefficient | Retirement immediately promoted leisure-time PA for both men and women, but did not affect the rate of change of PA for women. |
| Syse et al. (2017) | Norwegian  study on Life course, Ageing and Generation  (2002,2007) | 546  57-66 y (60.5 y)  47% | Multinomial multivariate  logistic regression: adjusted for age, gender, marital status, education,  public versus private sector employment, number of hours worked  per week,  stressful work situations, and sickness absence over the last 12 months | **Increased outdoor PA**  2.01 (1.16, 3.47)*  **Reduced outdoor PA**  0.83 (0.48, 1.44) | OR (95%CI) for retired people vs. working people | Retirees were more likely to increase PA. |
| Motegi et al. (2016) | Japanese Study of Aging and Retirement (2007,2009,2011) | 3,773  50+ y  (62.9y) | Basic pension eligibility age as IV: adjusted for age, age squared/100, married, number of children, logged income, poor health, depressed, IADL, mental stress at work physical stress at work | **Walking**  3.305*  **Light exercise**  Men: -0.194  Women: 0.407**  **Heavy exercise**  1.828* | Fixed effect IV coefficient | Retirees increased walking and exercise. |
| Stenholm et al. (2016) | Finnish Public Sector study  (2000-2002, 2004/2005, 2008/2009 and 2012/2013. | 9,488  79.4% | Linear regression analyses with generalized estimation equations. Adjusted for retirement age, sex, occupation, BMI, smoking, alcohol use, and number of chronic diseases | **Total weekly leisure–time PA (MET hours)**  1.81***  **Moderate-level PA (hours/week)**  0.58***  **Vigorous PA (hours/week)**  -0.08 | Mean change around statutory retirement | PA increased during 4-year retirement transition |
| Feng et al. (2016) | US Health and Retirement Survey (waves 7 to 10: 2004-2010) | 5,754  50-70 y | Logistic regression: adjusted for age, gender and the baseline year of survey | **Vigorous activities**  1.28 (1.09, 1.50)**  **Moderate activities**  1.24 (1.07, 1.43)** **Light activities**  1.10 (0.95, 1.28) | OR (95%CI) for retired people vs. working people | Retirement increased PA. |
| Ding et al., (2016) | Social, Economic, and Environmental Factor study in Australia (2006-2009) | 267,153  ≥45 y | Logistic regression: adjusted for follow-up time, age, sex, educational attainment, marital status, general self-rated health, area of residence (major city/regional/remote), and the baseline PA | **Insufficient PA:**  0.73 (0.65, 0.83)***  **Excessive sitting:** 0.34 (0.29, 0.39)*** | OR (95%CI) for retired people vs. working people | Retirement was associated with reduced odds of physical inactivity and excessive sitting. |
| Kämpfen and Maurer (2016) | US Health and Retirement Study (waves 7 to 10: 2004-2010) | 13,491  50-80 y (65.3 y)  57% | Early and normal retirement ages as IVs for retirement: adjusted for age, age^2^, gender, race, ethnicity, marriage, education, household wealth, number of children, smoking, drinking, physical demands of the current/longest held job, wave and interview date. | **Probability of meeting the US governments’ 2008 PA Guidelines:**  Men: 0.333*  Women: 0.392**  Men (Fixed): 0.328*  Women (Fixed): 0.227^ꝉ^ | IV coefficients  IV Fixed effects coefficients | Retirement increases the probability of meeting PA guidelines. |
| Eibich et al.  (2015) | German Socio-Economic Panel Study (1984-2004) | More than 20,000 individuals  55-70 y (61.7)  51.8% | Regression Discontinuity Design using eligibility ages (60 and 65) as exogenous variation in retirement: adjusted for age, gender, lived in East Germany in 1989, education, occupation physical and mental strain, and partner is retired. | **Probability of doing regular PA:** 0.107** | Coefficients of Regression Discontinuity Design | Retirement decreases the probability of regular PA. |
| Barnett et al.  (2014) | European Prospective  Investigation into Cancer and Nutrition -Norfolk study in the UK (1997–2007) | 3,334  45-79 y  52.0% | Multivariate: adjusted for age, BMI, change in marital status, PA or TV viewing time at baseline, incidence of chronic disease, alcohol consumption, smoking. | **Overall PA (MET h/wk)**  Non-manual social class  Men: −40.9  (−49.5, −32.5) ***  Women: −26.9  (−33.9, −19.9) ***  Manual social class  Men: −49.6  (−63.2, −35.9) ***  Women: −31.6 (−43.4, −19.7) *** | Coefficients of linear regressions | Compared with continued employment,  retirement was associated with a decline in overall  activity. The decline is bigger among those retired from manual social class. |
| Insler (2014) | US Health and Retirement Study (1992-2010) | 6,276  ≥50 y  (workers:59.4y; retirees: 66.4 y)  Workers:43%  Retirees:46% | Workers' self-reported probabilities of working past ages 62 and 65 as an IV for retirement,  adjusted for age, age^2^, gender, black, Hispanic, married, assets, debt, and education. | **Participant in vigorous physical**  **activity** **3 or**  **more times per**  **week**  Long-term: 0.227^ꝉ^  Short-term: 0.048 | Coefficients of IV analysis | Retirement increases vigorous activity only at the 10% significance level. |
| Menai et al. (2014) | SU.VI.MAX (Supplementation with Antioxidants and  Minerals) study in France (2001-2007) | 2,841  ≥45 y (57.3 y)  48.9% | Multivariate: adjusted for age, sex, educational level, smoking status, and occupational PA at baseline. | **Sedentary behaviour**  Retired: 8.4 (0.42) *  Not retired:  4.7 (0.35)  **Leisure PA**  Retired: 2.5 (0.18)*  Not retired:  0.7 (0.11) | Mean (SE) changes in hours spend per week | In subjects transitioning to retirement, both sedentary behaviour and PA during leisure-time increased substantially  over the 6-year follow-up period. The mean increase in total sedentary behaviour was about three times higher than the mean increase in leisure-time PA. |
| Turrell et al. (2014) | How Areas in Brisbane Influence Health and  Activity study in Australia (2007,2009, and 2011) | 9,577  40-65 y | Multilevel modelling: neighbourhoods (level 3), between-individuals (level 2),  and within-individuals (level 1).  Multivariate: adjusted for age, sex, year, neighbourhood disadvantage, education and household income. | **Minutes walking for transport**  White collar:  0.6 (−5.8, 7.0)  Blue collar:  3.0 (−5.2, 11.1)  Retired:  −16.0 (−24.0, −7.9)**  White collar × time: −3.4 (−9.5, 2.7)  Blue Collar × time:  −0.9 (−8.6, 6.9)  Retired × time:  −11.1 (−18.3, −4.0)** | Coefficients of multilevel mixed-effects linear regression, professionals as reference | Among the employed, there was no association between occupation and minutes walking for transport.  Compared with managers  and professionals, retired people walked on average 16 minutes less.  Average minutes of walking for transport declined for all socioeconomic groups;  however, the declines were steeper for the retired. |
| Koeneman, (2012) | Longitudinal Aging Study Amsterdam in the Netherlands (1992/1993 -1995/1996) | 186  >=55 y  36% | Gender, baseline activity, age, gender and educational. | **Moderate to vigorous PA**  32.5*** | Linear regression coefficient after ranking | Retirement was associated with increased moderate to vigorous PA. |
| Kuvaja-Kollner et al. (2012) | Eastern Finnish men and women | 1,410  57-78 y (Men: 66.3 y; Women: 66.6 y)  50.6% | Multivariate: adjusted for age, gender, income, education, and intervention dummies groups. | **Time spent on moderate-heavy physical exercise (hours/week)**  1.03*** | Coefficients of linear random effect | Retirement was associated with increased time spent on moderate-heavy physical exercise. |
| Sjösten et al. (2012) | GAZEL cohort (employees of French national  gas and electricity company, 2002- 2009) | 2,711  35-50 y  37% | Sex, age at retirement, marital status and occupational position. | **% walking at least 5 km/week**  Men: 36%  Women: 61% | Mean percentage | In both men and women, statutory retirement was associated with higher likelihood of walking at least 5 km/week. |
| Lahti et al. (2011) | City of Helsinki study in Finland (baseline in 2000-2002, follow up until 2007) | 6,706  40-60 y  81.3% | Baseline BMI, smoking, physical strenuousness of work, social economic position, and limiting longstanding illness. | **Change on time spent (min/week) in leisure PA**  Men: 42 (18-67)*  Women: 31 (18-44)*  **Physical inactivity:**  Men: 0.66 (0.43-1.04)  Women:  0.77 (0.60-0.99)* | Adjusted mean (95% CI)  OR of physical inactivity for retired people vs. working people (95% CI) | Retirement was associated with an increase in moderate-intensity leisure-time PA and a decrease in the proportion of inactive. |
| Touvier (2010) | SU.VI.MAX study in France (1998-2001) | 1,389  45-64 y (Women: 52.3 y  Men: 57.1 y)  49.7% | Age, education level and baseline value of the corresponding variable. | **Change in leisure PA (MET-h/week)**  Men  Working: -0.5  Retired: 8.1 ***  Women  Working: -1.9  Retired: 6.8***  **Change in time spent watching TV (min/day**)  Men:  Working: 15.0  Retired: 40.5***  Women:  Working:19.9  Retired: 33.5* | Mean | Retirement was associated with an increase in leisure-time PA and time spent on watching TV. |
| Brown et al. (2009) | Australian Longitudinal Study on Women's Health (2001-2004) | 8,762  51-56 y  100% | Multivariate: adjusted for area of residence and education, country of birth, household income, and weight change. | **Increasing leisure PA**  1.54 (1.24,1.91)*** | OR for retired people vs. non- retired people (95%CI) | Retirees were more likely to report an increase in leisure PA (compared to 3 years ago) than participants who were still employed |
| Chung et al. (2009) | US Health and Retirement Study (1996-2002) | 11,469  50-71 y  (60.3 y)  52.9% | Multivariate: adjusted for age, race/ethnicity education, marital status, household income, and current health problems. | **Total PA**  Physically demanding job: −0.075**  Sedentary job: 0.044** | Coefficients from fixed-effects regression | PA decreased with retirement from a physically demanding job but increased with retirement from a sedentary job. |
| Nekuda (2009) | US Health and Retirement Study (1998-2000) | 5,351  (59 y)  48.3% | Multivariate: adjusted for age, gender, education, income, race and perceived health status. | **Participant in vigorous physical**  **activity** **3 or**  **more times per**  **week**  1.06 (0.89,1.246) | OR for retired people vs. working people (95%CI) | Retirement is not associated with vigorous PA. |
| Henkens et al. (2008) | Panel study of retirement  in the Netherlands (2001-2007) | 1,604  50-64 y  (54.8 y)  25% | Multivariate: adjusted for gender, age, wealth, education and work environment in baseline. | **Increased leisure PA**  Voluntarily:  2.90(2.19-3.84)  Involuntarily:  2.14 (1.47-3.13)  **Decreased PA**  Voluntarily:  0.35 (0.22-0.56)  Involuntarily:  0.46 (0.23-0.92) | RR from logistic regression | People report more exercise after retirement, no matter retirement was perceived as voluntary or not. |
| Zheng (2008) | US Health and Retirement Study (1992-2004) | 6,935  50-73 y  43.2% | Ages of Social Security (62y) and Medicare (65y) eligibility as IVs for retirement: adjusted for age, age^2^, education, ethnicity, income, wealth, number of IADL/ADL limitations, self-rated health, ever being diagnosed cancer, diabetes, heart disease, hypertension, lung disease, and stroke. | **Participant in vigorous physical**  **activity** **3 or**  **more times per**  **week**  Men: 0.146 | Fixed effects IV coefficients | Retirement was not associated with vigorous PA. |
| Slingerland et al.  (2007) | GLOBE Study in the Netherlands (1991-2004) | 971  40-65 y  30% | Multivariate: adjusted for sex, age, marital status, chronic diseases,  and education | **Decline in work-related transport PA:**  3.03 (1.97, 4.65)***  **Decline in sports participation:**  0.66 (0.39, 1.10)  **Decline in non-sports leisure-time PA:**  0.36 (0.19, 0.68)*** | OR for retired people vs. working people (95%CI) | Retirement was associated with a significantly higher odds for a decline in PA from work-related transportation. |
| Berger et al. (2005) | West of Scotland Twenty-07 Study in the UK (1991-1995/1996) | 699  60 y  54.5% | Multivariate: adjusted for gender, social class, car ownership, education, smoking, deprivation score, diet, and self-rated health. | **Meeting PA recommendations** (**through**  **total life activity)**  2.72 (1.88, 3.97)* | OR for working people vs. retired people (95%CI) | Employed had higher odds of meeting PA recommendations with total PA compared to retired people. |
| Mein et al. (2005) | Whitehall II longitudinal study in the UK (phase 5) | 6,224  45-69 y 27.7% | Multivariate: adjusted for age, marital status, month of questionnaire completion, and employment grade. | **Meeting PA recommendations (through**  **total life activity)**  Partly retried (work < 30h/week)  Men:  2.65 (1.89-3.71) *  Women:  1.89 (1.07-3.34)  Fully retired  Men:  3.46 (2.78-4.30) *  Women:  2.53 (1.85-3.46)*  Men:  0.36 (0.19-0.69)*  Women:  0.53 (0.29-0.97)* | OR for retired people vs. working people (95%CI)  OR according to employment grade, lowest vs. highest (95%CI) | Fully or partly retired people are more likely to take the recommended amount of PA than working people, and this benefit is evident amongst those who retired from higher employment grades |
| Evenson et al. (2002) | US Atherosclerosis Risk in Communities Study cohort  (baseline: 1986-1989, follow up until 1993-1995) | 7,782  45-65 y  30% | Age, centre, education, and baseline perceived health status | **Sport plus leisure scores**  African-American women  Retired:  0.29 (0.17, 0.42)*  Working:  0.15 (0.05, 0.25)*  African-American men  Retired:  0.43 (0.26, 0.60)*  Working:  0.03 (−0.09, 0.15)  White women  Retired:  0.15 (0.07, 0.23)*  Working:  −0.05 (−0.11, 0.01)  White men  Retired:  0.20 (0.14, 0.26)  Working:  0.00 (−0.05, 0.05) | Adjusted mean (95%CI) | Participants who retired during follow-up were more likely to increase their sport participation and television watching than those who continued to work. |
| Midanik et al. (1995) | Kaiser Permanente Retirement Study in the USA (1985-1987) | 595  60-66 y  42.5% | Multivariate: adjusted for baseline mental health or health behaviour, age, gender, marital status, and education. | **Leisure regular exercise**  3.5 (2.0, 6.2)***  2.2 (1.2, 4.0)** | RR for retired people vs. not retired people (95%CI) | Retired members were  more likely to engage in regular exercise more often as compared to those who did not retire. |
| Patrick et al. (1986) | Volunteers from the steel industry and a light manufacturing industry in the UK | 72  ≥60 y  52.7% | No adjustment | **Time spend on total PA**  Pre-retirement:  Men: 91  Women: 89  Post-retirement:  Men:109*  Women: 62* | Mean (minutes/day), p-value from t-test | Retirement was associated with an increase in total PA in men and a decrease in women. |

OR: odds ratio. RR: risk ratio. *p<0.05; **p<0.01; ***p<0.001

**Table 2s.e** Longitudinal studies on retirement and smoking.

| **Author (year)** | **Data used** | **Sample size, age (mean), and % women** | **Type of analysis & covariates** | **Effects on outcome measure(s)** | **Effect type** | **Overview of findings** |
| --- | --- | --- | --- | --- | --- | --- |
| Kesavayuth et al. (2018) | Survey of Health Aging and Retirement in Europe (2004-2012; waves 1, 2, 4, 5) | 5,319  50-75y  48.7% | Eligibility  ages for early and normal retirement as IVs for retirement: adjusted for age, gender, household income, education, household size, marital status,  working in a physically  demanding job,  working in a stressful  job, European region and wave. | **Probability of smoking**  Retired: 0.052**  Retired × Baseline smoker: −0.400*** | Fixed effects IV coefficients | Non-smokers did not increase smoking upon retirement. Retirement led to less smoking for those who smoked before retiring. |
| Müller and Shaikh (2018) | Survey of Health Aging and Retirement in Europe (2004-2013; waves 1, 2, 4, 5) | 23,589  50 y+(63.35 y) | Age crossing the official retirement age and the interaction of that indicator with age as IVs | **Likelihood of smoking:**  0.05  **Number of cigarettes smoked per day:**  2.18 | Fuzzy regression discontinuity coefficients | Retirement had no effect on smoking. |
| Celidoni and Rebba  (2017) | Survey of Health Aging and Retirement in Europe (2004-2010; waves 1, 2, 4) | 13,464  45-85 y  (65.05 y)  46.3% | Changes in eligibility rules for early retirement and old-age pension across several European countries and over time as IVs for retirement: adjusted for gender, education, age, age^2^/100, marital status, wealth, number of grandchildren, country, and wave | **Probability of smoking**  White collar: -0.028  Blue collar: -0.084** | Fixed effects two-stage least squares IV coefficients | Those retired from physical demanding jobs were less likely to smoke when they retire, but not for those white collars. |
| Xue et al. (2017) | China Health and Nutrition Survey (1991–2011) | 1,084 (46.4 y)  (41.3%) | Piecewise regression: adjusted for  sex, education, province, spouse's working status/no spouse in the household, occupational skill level, PA level at the workplace, and per capita annual household income, alcohol consumption, and BMI age at retirement  age in 1991 | **Change of smoking status**  Light/moderate use versus nonuse:1.003 (0.965-1.042)  Heavy use versus non-use:  0.963  (0.923-1.005) | OR (95% CI) | No significant association with smoking |
| Oshio and Kan (2017) | The Longitudinal Survey of Middle-Aged and Older Adults in Japan (2005–2014) | 9,283  50-59 y  52.2% | Eligibility for public pension and Age−Eligibility age as IVs: adjusted for living along, have a spouse, provide informal care for family member and wave | **Current smoking**  Immediate impact of retirement:  Men: −0.009  Women: -0.047  Change in the rate of change after retirement:  Men: 0.132*  Women: -0.257 | Fixed effect IV coefficient | Retirement accelerated the rate of smoking cessation among men only. |
| Motegi et al. (2016) | Japanese Study of Aging and Retirement (2007,2009,2011) | 3,773  50+ y  (62.9y) | Basic pension eligibility age as IV: adjusted for age, age squared/100, married, number of children, logged income, poor health, depressed, IADL, mental stress at work physical stress at work | **Smoking frequency**  -0.329 | Fixed effect IV coefficient | Retirement has no effect on smoking frequency. |
| Ding et al., (2016) | Social, Economic, and Environmental Factor study in Australia (2006-2009) | 267,153  ≥45 y | Logistic regression: adjusted for follow-up time, age, sex, educational attainment, marital status, general self-rated health, area of residence (major city/regional/remote), and the baseline smoking | **Smoking**  0.74 (0.55, 0.99)* | OR (95%CI) for retired people vs. working people | Retirement reduces smoking. |
| Eibich  (2015) | German Socio-Economic Panel Study (1984-2004) | More than 20,000 individuals  55-70 y  (61.7 y)  51.8% | Regression Discontinuity Design using eligibility ages (60y and 65y) as exogenous variation in retirement: adjusted for age, gender, lived in East Germany in 1989, education, occupation physical and mental strain, and partner is retired. | **Probability of smoking:** -0.057* | Coefficients of Regression Discontinuity Design | Retirement decreases the probability of smoking. |
| Ayyagari (2014) | US Health and Retirement Study (1992-2008) | 11,576  60-80 y  (68.6 y)  46.4% | Eligibility age of Social Security  benefits (62y) as an IV for retirement: adjusted age, age^2^, gender, race, education, wave, and census division. | **Probability of smoking:**  0.225** | Marginal probability of smoking from IV analysis | Retirement increases the  probability of smoking among ever smokers |
| Insler (2014) | US Health and Retirement Study (1992-2010) | 6,276  ≥50 y  (Workers: 59.4 y; Retirees: 66.4 y)  Workers:43%  Retirees:46% | Workers' self-reported probabilities of working past ages 62 and 65 as an IV for retirement,  adjusted for age, age^2^, gender, black, Hispanic, married, assets, debt, and education. | **Smoking**  Long-term: -0.393*  Short-term: -0.273^ꝉ^ | Coefficients of IV analysis | Retirement exerts a beneficial  influence on quit smoking. |
| Henkens (2008) | A panel study on retirement behavior in the Netherlands, carried out  by the Netherlands Interdisciplinary Demographic Institute  (2001-2007) | 1,604  50-64 y  (54.8 y)  25% | Multivariate: adjusted for gender, age, wealth, education and work environment in baseline | **Risk of decreased smoking**  Involuntarily  0.50 (0.25, 0.99)*  Voluntarily  0.95 (0.66,1.38)  **Risk of increased smoking**  Involuntarily  3.68 (1.45, 9.30)**  Voluntarily  2.06 (0.74,5.79) | RR (95%CI) for retired people vs. working people | Voluntary retirement was not associated with smoking status.  The involuntarily retired had both higher risk  of increased smoking and lower risk of decreased smoking. |
| Lang et al. (2007) | Health  Survey for England  English Longitudinal Study of Ageing (1998-  2004) | 1,712  55-70y  55.4% | Multivariate: adjusted for age, gender, BMI, alcohol consumption, household wealth,  longstanding limiting disabilities, and education. | **Risk of quitting smoking**  2.50 (1.35-4.62)*  2.33 (1.24-4.38) | OR for retired vs. working people (95%CI)  OR for retired (retired for reasons of ill-health were excluded) vs. working people (95%CI) | Retired people were more likely to quit smoking as those who continued to work. Results were robust when those who retired for reasons of ill-health were excluded. |
| Midanik et al. (1995) | Kaiser Permanente Retirement Study in the USA (1985-1987) | 595  60-66 y  42.5% | Multivariate: adjusted for baseline mental health or health behaviour, age, gender, marital status, and education. | **Risk of smoking**  Men: 0.6 (0.2,2.1)  Women: 1.2 (0.3,4.9) | RR for retired people vs. not retired people (95%CI) | Retirement was not associated on smoking status. |
| Morris et al. (1992) | British Regional Heart Study (baseline: 1978-1980, follow up until 1983-1985) | 6,057  40-59 y  0% | Multivariate: adjusted for age, social class, and town of residence. | **Quit smoking**  Retired (not due to illness): 31.4  Working: 26.2 | % of people | No significant association between retirement and smoking. |

**Table 2s.f** Longitudinal studies on retirement and drinking.

| **Author (year)** | **Data used** | **Sample size, age (mean), and % women** | **Type of analysis & covariates** | **Effects on outcome measure(s)** | **Effect type** | **Overview of findings** |
| --- | --- | --- | --- | --- | --- | --- |
| Kesavayuth et al. (2018) | Survey of Health Aging and Retirement in Europe (2004-2012; waves 1, 2, 4, 5) | 5,319  50-75y  48.7% | Eligibility  ages for early and normal retirement as IVs for retirement: adjusted for age, gender, household income, education, household size, marital status,  working in a physically  demanding job,  working in a stressful  job, European region and wave. | **Probability of drinking**  Retired: 0.446*** | Fixed effects IV coefficients | Retirement increased drinking. |
| Müller and Shaikh (2018) | Survey of Health Aging and Retirement in Europe (2004-2013; waves 1, 2, 4, 5) | 23,589  50 y+  (63.35 y) | Age crossing the official retirement age and the interaction of that indicator with age as IVs | **Drinking frequency:**  1.21*** | Fuzzy regression discontinuity coefficients | Retirement increased drinking frequency |
| Celidoni and Rebba  (2017) | Survey of Health Aging and Retirement in Europe (2004-2012; waves 1, 2, 4) | 13,464  45-85 y  (65.05 y)  46.3% | Changes in eligibility rules for early retirement and old-age pension across several European countries and over time as IVs for retirement: adjusted for gender, education, age, age2/100, marital status, wealth, number of grandchildren, country, and wave. | **Probability of drinking every day:**  Men:0.074*  Women:0.006 | Fixed effects two-stage least squares IV coefficients | Men are more likely to drink every day when they retire, but not women. |
| Syse et al. (2017) | Norwegian  study on Life course, Ageing and Generation  (2002, 2007) | 546  57-66 y (60.5 y)  47% | Multinomial multivariate  logistic regression: adjusted for age, gender, marital status, education,  public versus private sector employment, number of hours worked  per week,  stressful work situations, and sickness absence over the last 12 months. | Increased alcohol intake  2.03* (1.28, 3.21)  Reduced alcohol intake 1.87* (1.10, 3.17) | OR for retired people vs. working people (95%CI) | Retirement is associated with both an increase and a reduction in alcohol  intake. |
| Xue et al. (2017) | China Health and Nutrition Survey (1991–2011) | 1,084 (46.4 y)  41.3% | Piecewise regression: adjusted for  sex, education, province, spouse's working status/no spouse in the household, occupational skill level, PA level at the workplace, and per capita annual household income, smoking, BMI, age at retirement, and  age in 1991. | **Change of drinking status**  Light/moderate use versus nonuse:1.005(0.957-1.057)  Heavy use versus nonuse:  0.919**  (0.862-0.982) | OR for retired people vs. working people (95% CI) | Retirement was accompanied by a reduction in the probability of being a heavy alcohol drinker. |
| Oshio and Kan (2017) | The Longitudinal Survey of Middle-Aged and Older Adults in Japan (2005-2014) | 9,283  50-59 y  52.2% | Eligibility for public pension and Age−Eligibility age as IVs:adjusted for living along, have a spouse, provide informal care for family member and wave. | **Heavy drinking:**  Immediate impact of retirement:  Men: −0.026  Women:-0.019  Change in the rate of change after retirement:  Men: 0.578  Women:-0.231 | Fixed effect IV coefficient | Retirement was not associated with heavy drinking. |
| Motegi et al. (2016) | Japanese Study of Aging and Retirement (2007,2009,2011) | 3,773  50+ y (62.9y) | Basic pension eligibility age as IV: adjusted for age, age squared/100, married, number of children, logged income, poor health, depressed, IADL, mental stress at work and physical stress at work. | **Grams of alcohol consumed per**  **day**  **-1.803**** | Fixed effect IV coefficient | Retirement decreases alcohol consumption |
| Ding et al., (2016) | Social, Economic, and Environmental Factor study in Australia (2006-2009) | 267,153  ≥45 y | Logistic regression: adjusted for follow-up time, age, sex, educational attainment, marital status, general self-rated health, area of residence (major city/regional/remote), and the baseline drinking. | **Excessive alcohol use**  **1.17 (1.00, 1.36)** | OR for retired people vs. working people (95%CI) | Retirement was not associated with excessive drinking. |
| Eibich  (2015) | German Socio-Economic Panel Study (1984-2004) | More than 20,000 individuals  55-70y (61.7)  51.8% | Regression Discontinuity Design using eligibility ages (60 and 65) as exogenous variation in retirement: adjusted for age, gender, lived in East Germany in 1989, education, occupation physical and mental strain, and partner is retired. | **Probability of drinking regularly:** 0.048  **Probability of no drinking:** -0.116*  **Probability of having health conscious diet:** 0.056 | Coefficients of Regression Discontinuity Design | Retirement decreases the probability of abstaining from alcohol. No significant impact of retirement on the probability of regular alcohol consumption or following health conscious diet was found. |
| Iparraguirre  (2015) | English Longitudinal  Study of Ageing (wave 4-5) | 9,251  50-89 y  (66.6 y)  55.5% | Multivariate: adjusted for age, marriage, caring responsibilities, number of children inside the household, economic activity, social detachment, and healthy diet. | **From not at higher risk to higher risk drinker:**  Men: OR=1.298  (0.891, 1.890)  Women: OR=1.131 (0.793, 1.614)  **From higher risk drinker to not at higher risk:**  Men: OR=1.017  (0.707, 1.465)  Women: OR=1.28 (0.936, 1.750) | Coefficients of logistic regression (retired people vs. working people)  Transition among retired people, OR from Markov chain models (95%CI) | Being retired at baseline (wave 4) is not associated with the transition between not being at a higher risk to becoming a higher risk drinker, and vice versa. |
| Tamers et al. (2014) | GAZEL cohort (employees of French national  gas and electricity company, 1992 - 2008) | 20,625  35-50 y  23.6% | Multivariate: adjusted for age, self-rated health, education and employment grade. | **Risk of heavy alcohol consumption**  Men  *Year -1 vs. -5:*  1.15 (1.08, 1.23)***  *Year +1 vs. -1:*  1.38 (1.32, 1.47)***  *Year 0:*  1.21 (1.15, 1.27)***  *Year +5 vs. +1:*  0.869 (0.83, 0.92)***  Women  *Year -1 vs. -5:*  1.23 (0.99, 1.54)^ꝉ^  *Year +1 vs. -1:*  1.34 (1.11, 1.62)**  *Year 0:*  1.19 (1.01,1.40)*  *Year +5 vs. +1:*  0.877 (0.72,1.07) | OR for before, during, after, and at the time of retirement | For men, heavy alcohol consumption increased in the years up to, surrounding retirement, and at the time of retirement, and then decreased after retirement.  Women follow the similar pattern, but the increase of heavy alcohol consumption before retirement and the decrease after retirement did not reach a 0.5% significance level. |
| Wang et al.  (2014) | US Health and Retirement Study (seven waves 1995- 2008) | 4,674  >50 y  45.2% | Multivariate: adjusted for race/ethnicity, education, age, marital status, non-housing wealth, depressive symptoms score, number of medical conditions and alcohol consumption at baseline. | **Average weekly alcohol consumption**  Among participants who reported alcohol use at follow-up  Men:  1.14 (−0.10, 2.38)  Women:  −0.33 (−1.50, 0.85)  Among participants who reported alcohol use at both baseline and follow-up  Men:  1.90 (0.43, 3.36)*  Women:  −0.33 (−1.90, 1.25) | Coefficients of linear regressions | Retirement is associated with higher weekly alcohol consumption for men who reported drinking at both follow-up and the baseline (i.e. consistent drinkers). No association was observed among women. |
| Bobo et al. (2013) | US Health and Retirement Study (1998-2008 | 3,105  50-65 y  0% | Multivariate: adjusted for baseline age, race, ethnicity, education, marital status, smoking, binge drinking, vigorous exercise, BMI, depression, pain, self-reported health, and chronic disease. | **Infrequent/ non-drinkers to follow an increasing consumption trajectory**  0.86 (0.53,1.40)  **Regular drinkers at baseline to follow a decreasing trajectory**  1.18 (0.73,1.91)  **Highest alcohol intake at baseline to follow a decreasing trajectory**  1.48 (0.83,2.64) | OR for working people vs. fully retired people (95%CI) | Retirement is not associated with of the trajectories of alcohol consumption among men. |
| Bobo et al. (2011) | US Health and Retirement Study (1998-2008) | 4,439  50-65 y  100% | Multivariate: adjusted for baseline age, race, ethnicity, education, marital status, smoking, binge drinking, vigorous exercise, BMI, depression, pain, self-reported health, and chronic disease. | **Increasing drinkers vs. non/infrequent drinkers**  1.53 (0.97,2.40)  **Decreasing drinkers vs. stable drinkers**  0.63 (0.41,0.98)* | OR for working people vs. fully retired people (95%CI) | Retired women were more likely to become decreasing drinkers. |
| Zins et al. (2011) | GAZEL cohort in France (1992 - 2007) | 12,384  50-63 y  19% | Multivariate: adjusted for time window, birth cohort, SES category and the interaction ‘year × SES category’. | **Prevalence of heavy drinking 1 year before to 1 year after retirement**  Men: Increase by 1.3% to 3.1% according to occupations.  Women: Increase by 3.3% to 6.6%  **Prevalence of heavy drinking 1 to 5 years after retirement**  Men: Decrease by 0.4% to 2.8%  Women: Decrease by 0% to 2.4% | % of change | Retirement increase the risk of excessive alcohol consumption, temporarily (1 year before and after retirement) in most people and permanently in the small group of women managers |
| Brennan et al. (2010) | A 10-year longitudinal study of late-life drinking behaviour in the USA | 595  (62 y)  44% | Multivariate: adjusted for baseline age, gender, income, health, and problem drinker status. | **Quantity of alcohol consumed**  0.29  **Frequency of alcohol consumption**  0.14 | Slopes of trajectories | Retirement status cannot predict frequency or quantity of alcohol consumption. |
| Platt et al. (2010) | US Health and Retirement Study (1992-2006) | 6,787  51-61 y  54.7% | Multivariate: adjusted for baseline characteristics and changes in demographic characteristics, health,  and social support that occurred during the observation  period. | **Increasing drinker** 0.974 (0.636, 1.492)  **Decreasing drinker**  0.990 (0.811, 1.208) | OR for retired people vs. not retried (95%CI) | Retirement was not associated with drinking behaviour. |
| Henkens (2008) | Netherlands  (2001-2007) | 1,604  50-64 y  25% | Multivariate: adjusted for gender, age, wealth, education and work environment in baseline. | **Risk of decreased alcohol use**  Involuntarily  0.47 (0.29,0.73)**  Voluntarily  0.72 (0.51,1.01)  **Risk of increased alcohol use**  Involuntarily  0.48 (0.19, 1.20)  Voluntarily  1.36 (0.80, 2.34) | RR for retired people vs. working people from logistic analyses | Voluntary retirement was not associated with alcohol consumption status.  The involuntarily retired had lower risk of decreased alcohol use. |
| Perreira and Sloan (2001) | US Health and Retirement Study (1992-1998, 4 waves) | 7,731  51-61 y  49.5% | Multivariate: adjusted for social support/coping skills, baseline socioeconomic characteristics: annual household  income; years of education; race/ethnicity,  religious affiliation, age, gender, and marital status. | **Increased drinking**  Retired:  1.6 (1.3, 2.1)**  **Decreased drinking**  Retired*problem drinking:  0.7 (0.5-1.0)* | OR for retired people vs. working people (95%CI) | people who experienced retirement in recent years were more likely to increase drinking, and people who had a history of problem drinking were less likely to decrease drinking if they had experienced retirement. |
| Bacharach et al. (2004) | A cohort of blue collar males in the USA (2000-2002) | 307  43-70 y  (57 y)  0% | Multivariate: adjusted for baseline age, marital status, and health status. | **Drinking frequency**  0.003  **Drinking quantity**  0.03  **Problem drinking**  0.005  **Currently drinking**  -0.24  **Periodic heavy drinking**  0.70* | Coefficients from logistic regression | Retirement generally heralds no great shift in alcohol consumption or drinking behaviours, but individuals opting to retire fully were twice as likely to engage in periodic heavy drinking as those continuing to work. |
| Gee et al. (2007) | National longitudinal study of  health and aging in Japan (1987-1999) | 2,566  60- 96 y (68 y)  54.4% | Multivariate: adjusted for age, gender, and education.  cognitive impairment, social support, depression  and demographics. | **Declined trajectory**  (drank the most at age 60, but exhibited the sharpest decline over age)  1.65**  **Stable trajectory**  1.14  **Curvilinear trajectory**  1.20 | RRR for not working people vs. working people (95%CI) | Alcohol consumption dropped significantly with not working people |
| Rodriguez & Chandra (2006) | National Survey of Families and Households in the USA (1987/88 -  1991/92) | 7,599  ≥16 y  60% | Multivariate: adjusted for age, race/ ethnicity, marital status and partnership stability,  years of education, total household income, total assets  value, number of children in the household, satisfaction with relationships  with friends and family, having a mental or physical condition, weeks unemployed and looking for work in 1991, having  alcohol problems in 1987, unemployment rates in 1992, and type of residence area. | **≥ 5 drinks at one time vs. no drinking**  Men:  0.57 (0.33, 0.96)*  Women: 1.74 (0.88, 3.29)  **< 5 drinks at one time vs. no drinking**  Men:  1.30 (0.91, 1.85)  Women:  1.15 (0.85, 1.55)  **≥5 at one time vs. drinking less**  Men:  0.40 (0.23, 0.71)*  Women:  1.37 (0.69, 2.69)  **≥30 drinks in past 30**  **days vs. drinking**  **< 30 drinks**  Men:  0.58 (0.35, 0.93)*  Women:  1.55 (0.98, 2.45) | OR for retired people vs. full time working people (95%CI) | Retired men were more likely to drink less, but not retired women. |
| Neve et al. (2000) | Participants of the Dutch province of  Limburg (1980-1989) | 1,980  16-64 y | Multivariate: adjusted for age, gender, and education. | **Average Alcohol Consumption**  Men: -2.9  Women: +0.05  **Drinking Problems**  Men: -0.3  Women: -0.1 | Change of % people | Retirement was associated with a decrease in alcohol consumption and alcohol-related problems. |
| Midanik et al. (1995) | Kaiser Permanente Retirement Study in the USA (1985-1987) | 595  60-66 y  42.5% | Multivariate: adjusted for baseline mental health or health behaviour, age, gender, marital status, and education. | **Heavy drinking**   1. (0.5, 2.2)   0.8 (0.3, 2.3)  **No alcohol problems**   1. (0.5, 2.0)   2.8 (1.0, 8.0)*  **Frequency of drunkenness**  0.6 (0.3,1.2)  0.7 (0.2,2.2) | RR for retired people vs. not retired people (95%CI) | Retired women were more likely to report no alcohol problems as compared to  non-retired women. There were no differences between the groups on alcohol consumption, and frequency of drunkenness. |
| Morris et al. (1992) | British Regional Heart Study (baseline: 1978-1980, follow up until 1983-1985) | 6,057  40-59 y  0% | Multivariate: adjusted for age, social class, and town of residence. | **Drinkers reduce drinking:**  Retired (not due to illness): 37.9  Working: 34.9 | % of people | No significant association between retirement and decreased drinking. |
| Ekerdt et al. (1989) | Veterans  Administration Normative Aging Study (1981-1983) | 416  0% | Multivariate: adjusted for baseline age, marital status, occupational prestige and follow-up reports of decreased drinking due to health. | **Onset of periodic heavier drinking**  Working: 3.2  Retired: 8.5*  **Onset of problems with drinking**  Working: 2.8  Retired: 9.0* | % change from T1 to T2. | Retirement generally heralds no great shift in alcohol  consumption or drinking behaviors. However, retirees were  more likely to report the onset of periodic heavier drinking and problems with drinking. |
| Glynn et al.  (1988) | Normative Aging Study in the USA (1982-1984) | 1,556  39-92 y (60.1y)  0% | Multivariate: adjusted for age, type A score, social status, and marital status. | **Non-drinking**  0.0107  **Drinking 3 or more drinks per day**  0.324  **Problems with drinking**  0.186  **Periodic heavier drinking**  0.078 | Coefficient from logistic regression | Retirement was not related alcohol consumption or drinking behaviour. |

**Table 2s.g** Longitudinal studies on retirement and diet.

| **Author (year)** | **Data used** | **Sample size, age (mean), and % women** | | **Type of analysis & covariates** | **Effects on outcome measure(s)** | **Effect type** | | **Overview of findings** |
| --- | --- | --- | --- | --- | --- | --- | --- | --- |
| Hassen et al., (2017). | NutriNet-Santé cohort in France (2009–2014 or 2010–2015) | | 577  50-64y  69.7% | Mixed model: adjusted for total energy intake with random effects of the time and the period | **Adherence to nutritional recommendations**  Women: -0.4***  Men: -0.2  Changes of dietary intakes with retirement were particularly marked in men with the lowest income at baseline (did not show coefficients) | Coefficients | Transition to retirement was associated with unhealthier dietary intakes. | |
| Ding et al., (2016) | Social, Economic, and Environmental Factor study in Australia (2006-2009) | | 267,153  ≥45 y | Logistic regression: adjusted for follow-up time, age, sex, educational attainment, marital status, general self-rated health, area of residence (major city/regional/remote), and the baseline fruit and vegetable consumption | **Insufficient fruit and vegetable consumption** 0.92 (0.83, 1.02) | OR (95%CI) | Retirement was not associated with fruit and vegetable consumption | |
| Helldán et al. (2012) | Helsinki Health Study cohort in Finland (baseline: 2000-02, follow-up: 2007) | | 2,428  55-60 y  77% | Baseline food habits, marital status, occupational class, household income, limiting long-standing illness, BMI, PA, smoking | **Healthy food habits**  Men:  1.11 (0.73,1.70)  Women:  1.32 (1.08,1.62) | OR for retired people vs. working people (95%CI) | Transition to old age retirement led to healthier food habits among women. For men, similar trends could not be confirmed. | |
| Lauque et al. (1998) | A two-year prospective study on diet in France | | In French | In French | In French | In French | The amount of dietary nutrients consumed remained the same before and after retirement. | |

**Table 2s.h** Longitudinal studies on retirement and blood biomarkers.

| **Author (year)** | **Data used** | **Sample size, age (mean), and % women** | **Type of analysis & covariates** | **Effects on outcome measure(s)** | **Effect type** | **Overview of findings** |
| --- | --- | --- | --- | --- | --- | --- |
| Behncke (2012) | English Longitudinal Study of Ageing (2002-2006) | 1,439  >=50 y  50.5% | 1. State retirement age (65 years) as an IV for retirement: adjusted for age, gender, job characteristics, anticipation to stay in work, and pre-retirement health  2. Propensity score matching | **High CRP** **(>3 mg/L)**  IV: 0.149  Matching: 0.086  **High fibrinogen (>7 mmol/L)**  IV: 0.124  Matching:0.079*  **Low haemoglobin (<12 g/dL)**  IV:-0.007  Matching: 0.016 | Coefficients from non-parametric IV and non-parametric matching methods. | Retirement people had significantly higher CVD incidence than working people. |

1. ‘/’ after an index term indicates that all subheadings were selected.

   ‘exp’ before an index term indicates that the term was exploded.

   ‘.tw.’ indicates a search for a term in title/abstract.

   ‘.mp.’ indicates a free text search for a term.

   ‘$’ at the end of a term indicates that this term has been truncated.

   ‘? ‘in the middle of a term indicates the use of a wildcard.

   ‘adj’ indicates a search for two terms where they appear adjacent to one another. [↑](#footnote-ref-1)
2. ‘*’ in the middle of a term indicates the use of a wildcard, and at the end of a term indicates that this term has been truncated. [↑](#footnote-ref-2)
